# The Food provision, cUlture and Environment in secondary schooLs (FUEL) study: protocol of a mixed methods evaluation of national School Food Standards implementation in secondary schools and their impact on pupils’ dietary intake and dental health

**DOI:** 10.1136/bmjopen-2020-042931

**Published:** 2020-10-16

**Authors:** Marie Murphy, Miranda Pallan, Emma Lancashire, Rhona Duff, Ashley J Adamson, Suzanne Bartington, Emma Frew, Tania Griffin, Kiya L Hurley, Jayne Parry, Sandra Passmore, Vahid Ravaghi, Alice J Sitch, Suzanne Spence, Maisie K Rowland, Scott Wheeldon, Peymane Adab

**Affiliations:** 1 Institute of Applied Health Research, University of Birmingham, Birmingham, UK; 2 Human Nutrition Research Centre, Population Health Sciences Institute, Newcastle University, Newcastle upon Tyne, Tyne and Wear, UK; 3 Department for Health, University of Bath, Bath, UK; 4 Cancer Research UK Clinical Trials Unit, University of Birmingham, Birmingham, UK; 5 Services for Education Ltd, Birmingham, UK; 6 School of Dentistry, University of Birmingham, Birmingham, UK; 7 NIHR Birmingham Biomedical Research Centre, University Hospitals Birmingham NHS Foundation Trust, Birmingham, UK; 8 Wheelers Lane Technology College, Birmingham, UK

**Keywords:** epidemiology, public health, nutrition & dietetics

## Abstract

**Introduction:**

Excess free sugar intake is associated with obesity and poor dental health. Adolescents consume substantially more free sugar than is recommended. National (UK) School Food Standards (SFS) are in place but are not mandatory in all schools, and their impact on the diets of secondary school pupils is unknown. We aim to evaluate how SFS and wider healthy eating recommendations (from the national School Food Plan (SFP)) are implemented in secondary schools and how they influence pupils’ diets and dental health.

**Methods and analysis:**

Secondary-level academies/free schools in the West Midlands, UK were divided into two groups: SFS mandated and SFS non-mandated. Using propensity scores to guide sampling, we aim to recruit 22 schools in each group. We will compare data on school food provision and sales, school food culture and environment, and the food curriculum from each group, collected through: school staff, governor, pupil, parent surveys; school documents; and observation. We will explore the implementation level for the SFS requirements and SFP recommendations and develop a school food typology. We aim to recruit 1980 pupils aged 11–15 years across the 44 schools and collect dietary intake (24-hour recall) and dental health data through self-completion surveys. We will compare free sugar/other dietary intake and dental health across the two SFS groups and across the identified school types. School type will be further characterised in 4–8 case study schools through school staff interviews and pupil focus groups. Evaluation of economic impact will be through a cost-consequence analysis and an exploratory cost–utility analysis.

**Ethics and dissemination:**

Ethical approval was obtained from the University of Birmingham Ethical Review Committee (ERN_18-1738). Findings will be disseminated to key national and local agencies, schools and the public through reports, presentations, the media and open access publications.

**Trial registration number:**

ISRCTN 68757496 (registered 17 October 2019).

**Strengths and limitations of this study**

- This research fills a gap in the literature by evaluating the impact of national school food policy on the dietary intake of secondary school pupils in the UK.
- A validated online dietary assessment tool, adapted for use in an ethnically diverse population, will be used with 1980 secondary school pupils across 44 schools.
- The research will assess variation in the implementation of the School Food Standards, School Food Plan recommendations and other contextual factors across schools.
- The study design includes qualitative research to provide an in-depth understanding of school food provision, environments and the culture/ethos relating to this.
- The study aims to compare schools that are mandated with schools that are not mandated to adhere to the School Food Standards. These two groups may differ in other ways, so to improve the comparability of the two groups, a sampling approach based on propensity scores has been conducted.

## Introduction

Excess sugar consumption is a major contributor to increased energy intake/obesity, adverse cardiometabolic health^1^ and poor dental health.^2^ UK adolescents consume three times their recommended amount of total energy intake from free sugars^3^; almost half of 15- year-old individuals have dental caries^2^ and nearly a third have excess weight.^4^ Adolescence is an important time for dietary intervention as it is a key period for establishing dietary patterns,^5^ with greater autonomy over dietary decisions. A large proportion of adolescent dietary intake occurs while at school, making these opportune settings for intervention.

In the UK, a longstanding strategy for improving children’s diet has been nutritional standards for school food. School meal standards were first introduced in 1941, but fell out of favour later in the 20th century. In 2006, following a national school meal review,^6^ national School Food Standards (SFS) were relaunched in England and became a legal requirement for most state schools. In 2015, a Department for Education (DfE) SFS review resulted in substantial changes. For ease of implementation, nutrient-based standards were removed while retaining food-based standards underpinned by a nutrient framework.^7^

In addition to the national SFS, the School Food Plan (SFP) was launched in 2013, providing a wider set of non-statutory recommendations for schools that promote a ‘whole school’ approach to healthy eating.^8^ One central aim was to increase school meal uptake, as higher demand enables better quality meals to be served at a lower cost. Another aim was to provide practical support, advice and information for headteachers to help improve the quality and uptake of their school’s food. In addition to school food provision, the plan addresses how healthy eating can be incorporated within all aspects of school life and the wider community.^9^

Evaluation of the impact of the 2006 SFS on food provision and consumption has been conducted in primary (age 4–11 years) and middle (age 9–12 years) schools in the UK. Pre-SFS and post-SFS implementation comparisons showed improvements in overall dietary intake in pupils aged 4–7 years, but not in those aged 11–12 years.^10–12^ SFS implementation was good in primary schools but less so in middle schools.^10^ Evidence of SFS impact on secondary school pupils’ (aged 11–16 years) dietary intake is more limited. A study in 80 secondary schools compared school food provision in 2011 with that in 2004. They reported improvements in the nutritional content of school-provided food (in particular reduced confectionery availability) and pupils’ lunchtime food consumption, but did not examine total dietary intake.^13^

To date, the impact of the updated SFS or the SFP has not been evaluated. The way in which SFS legislation was introduced in England means that certain school types (including academies and free schools; 70% of secondary schools in England)^14^ that were set up between January 2010 and May 2014 are exempt from this legislation (although they can choose to voluntarily sign up to the standards), whereas schools of these types established before or after these dates are legally required to meet the SFS. This provides an opportunity to examine how a legal requirement to meet the SFS influences schools and their pupils. Given the lack of SFS/SFP evaluation in secondary schools, we aim to investigate their influence in these settings by comparing school food provision, sales, wider school factors related to food, pupil dietary intake and dental health across those mandated and not mandated to adhere to the current SFS. We will also explore variation in the implementation of the SFS and SFP recommendations and their economic impact.

## Methods and analysis

This is an observational, mixed-method design study, consisting of two phases. Phase I involves collecting a variety of data on SFS/SFP implementation, and school and pupil outcomes in the SFS mandated and non-mandated schools. We will compare outcomes across the two groups and develop a typology of schools based on SFS/SFP implementation and wider school contextual factors. In phase II, we will identify a small number of ‘case study’ schools and conduct a qualitative inquiry to further develop the school typology. Furthermore, we will undertake an economic evaluation to assess how the costs and outcomes vary by school type.

### Study setting

The sampling frame comprises secondary phase academies/free schools providing education to children aged 11–16 years and located within 14 Local Authority areas in the West Midlands region, UK. This region has a population of five million (21% of an ethnicity other than white British),^15^ urban, suburban and rural areas and areas of high socioeconomic deprivation.^16^ Other school types and academies that provide specialist alternative education have been excluded.

### Phase I

#### Sampling

To increase the comparability of the two school groups, stratified sampling, based on propensity scores, was used.^17^ We obtained routine data from the DfE on several characteristics for all schools in the sampling frame: Local Authority area; establishment type; urban/rural; total pupil roll size; Income Deprivation Affecting Children Index; inclusion of a sixth form; selective/non-selective admissions policy; religious affiliation/secular; and proportion of: male/female pupils; pupils from Black, Asian and ethnic minority groups; students with English as a foreign language; students eligible for free school meals; and pupils with Special Educational Needs. We developed propensity scores using linear regression with the SFS status of the school (mandated/non-mandated) as the outcome and school characteristics as explanatory variables. Propensity score quartiles were then used to create four groups with subsequent division by SFS status (based on the date they received academy/free school status), resulting in eight distinct sampling groups. Following the random ordering of each group, schools will be invited sequentially to participate, aiming to recruit five or six schools from each group.

Within participating schools, pupils in one class from each of years 7, 9 and 10 (aged 11–12, 13–14 and 14–15 years, respectively) will be invited to take part. Preference will be given to classes not streamed by academic ability or subject to enable participation of classes that are representative of the year group characteristics. There are no pupil exclusion criteria.

#### Sample size calculation

We used data on free sugar intake pre-SFS and post-SFS implementation from the study undertaken in middle schools by Adamson *et al*^10^ to inform our sample size calculation. Assuming an intraclass correlation coefficient of 0.1^18^ and balanced cluster sizes, we estimated that to detect a difference in mean free sugar intake at lunch of 4 g (20 g vs 16 g) between the two school groups, assuming an SD of 11 with 90% power and at 5% significance, we require 990 evaluable participants and 22 clusters (conservatively using schools^19^) in each group (total schools=44; total participants n=1980; cluster size=45).

#### Recruitment

Headteachers will be invited by post and email with a telephone follow-up. In participating schools, a liaison staff member will be identified and a contract, outlining expected commitments from both parties, will be signed. Once data collection is complete, participating schools will receive a school-specific summary report and £300.

At least 7 days prior to pupil data collection, pupils from the three selected classes will receive a participant information pack (comprising pupil and parent information sheets and a parental opt-out consent form). Schools will also be asked to email documents directly to parents. Pupils whose parents do not return a completed opt-out consent form will be invited to take part in the data collection sessions during the school day and asked for their electronic assent for study participation. Parents of pupils in selected classes will be invited to complete a self-administered parent survey (online or paper copy). Both pupils and parents who participate will receive a £5 voucher.

Participating schools will be asked to identify key staff/governors with roles relating to food provision, eating environment, food curriculum, or SFS/SFP implementation. Identified staff will be sent a staff participant information sheet (email/paper copy) and study invitation for a self-administered survey (online or paper format). Recruitment and data collection processes are outlined in the online supplemental file 1.

10.1136/bmjopen-2020-042931.supp1 Supplementary data

School and pupil recruitment commenced in October and November 2019, respectively, and was due to be completed in the 2019/2020 academic year. However, due to restrictions in place in England during the COVID-19 pandemic, recruitment and data collection were suspended in March 2020 and will recommence when schools reopen for all pupils to attend. All further data collection activities will be undertaken in accordance with the relevant Department of Health and Social Care and DfE’ health protection guidance.

#### Data collection

##### Data capture on SFS and SFP implementation and the wider school context

**Figure 1:**
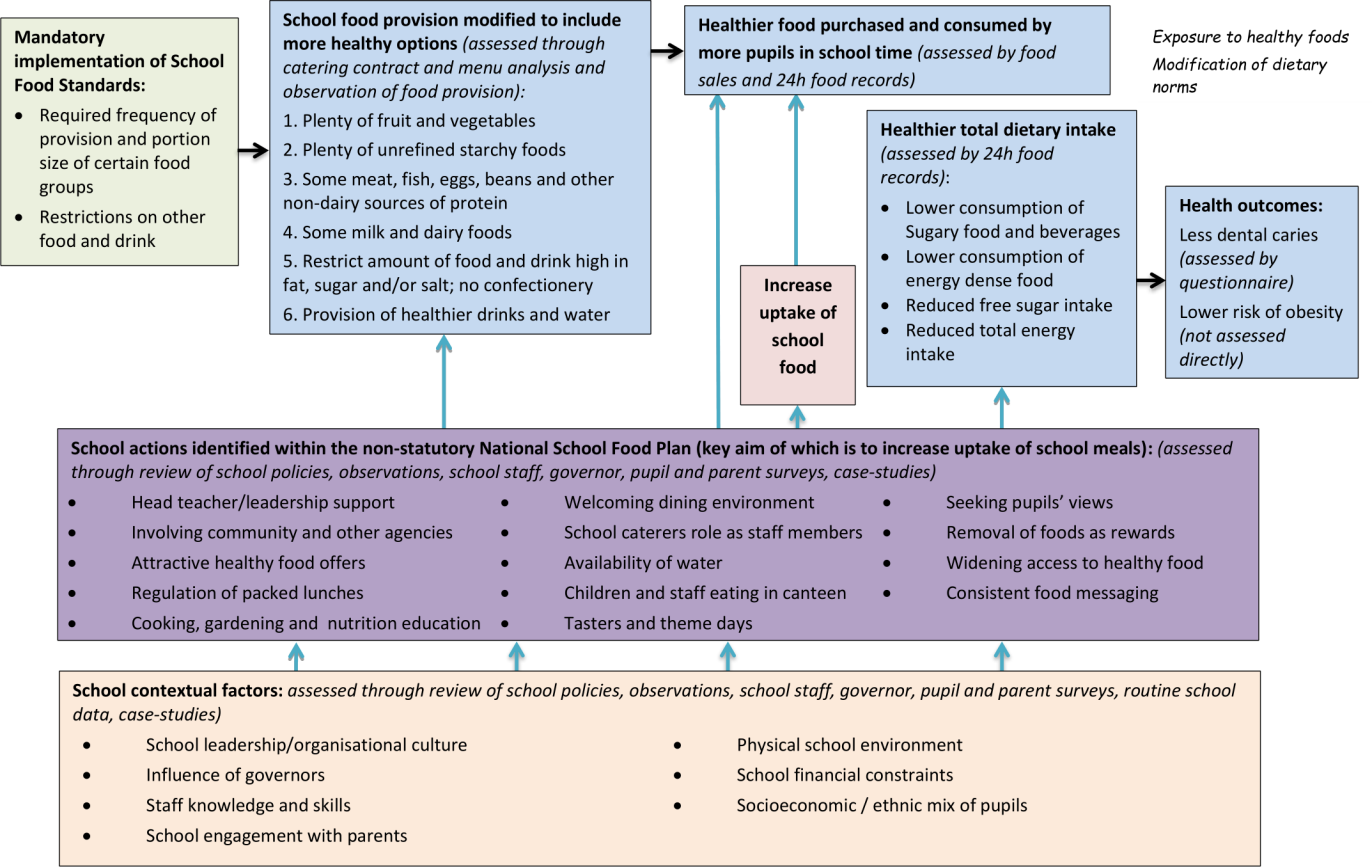
Logic model and theory of change for the influence of School Food Standards on children’s dietary intake and health outcomes.

A logic model setting out the processes by which the SFS/SFP are assumed to generate pupil health gains has been developed (figure 1). Briefly, we hypothesise that health gain materialises directly via a change in school food consumption and indirectly by curricular and other activities designed to change pupils’ dietary knowledge, attitudes and beliefs that impact on food consumption in and out of school. The extent to which the SFS/SFP achieve these health gains depends on their implementation within a school, which is influenced by key contextual factors (eg, school management, parental engagement and so on).

Our data collection strategy, guided by the principles for process evaluation outlined in the UK Medical Research Council guidance,^20^ will provide data to populate and refine the logic model. This will enable us to assess the extent to which the SFS requirements and SFP recommendations are embedded in each school and the relative importance of contextual factors in influencing this. Our data collection methods include: school menu analysis; observation of school food provision and the related environment; school document review; collection of aggregated routine data on pupil characteristics; and self-administered surveys for staff, governors, pupils and parents.

##### Assessment of SFS compliance and SFP recommendations implementation

SFS compliance assessment is based on the national SFS checklists for school lunch and school food other than lunch^21^ that consist of daily, weekly and three-weekly criteria for food and drink offered. Using an observation tool developed to measure compliance with the SFS daily criteria, trained researchers will record food and drink provision at all eating occasions across one school day, and at all school food outlets and dining areas. Compliance with the SFS weekly and three-weekly criteria will be assessed from schools’ weekly menus.

Implementation of the SFP recommendations is based on three guidance documents produced by the DfE: (1) a headteacher checklist; (2) a guide to creating a culture and ethos of healthy eating; and (3) guidance for governors.^22–24^ Implementation will be assessed through questions in the surveys to pupils, parents, staff and governors; researcher observation of food outlets and dining areas; and school document review, for example, school food policy, catering contract, curriculum documents and minutes of the Board of Governors and School Council meetings.

The school staff and governor surveys include a series of questions based on Normalisation Process Theory (NPT),^25^ to explore the implementation and sustained embedding of the SFS/SFP. The items in the NoMAD instrument (developed to assess the four NPT constructs)^26^ were adapted for relevance to the SFS/SFP and the school setting.

##### Assessment of school lunch uptake and food sales

To assess school lunch uptake, schools will be asked to provide their routinely collected school meal uptake data. To assess school food sales, we will request weekly aggregated food sales data for two prespecified months in the previous year. Most schools use online payment management systems, which provide sales data by types of food/drinks sold. We will extract data on the number of items sold for both SFS restricted food/drinks (including sugar-sweetened beverages, confectionery, fried food, snacks and so on) and predefined healthy foods and compare sales of these food categories across schools, taking into account school size and school lunch uptake.

##### Assessment of school contextual factors

We will collect data on a variety of contextual factors through observation; school document review; and the staff, governor, pupil and parent surveys, with further exploration in the second phase case studies. These factors include: the physical school environment; school leadership and organisational culture; the influence of governors; staff knowledge and skills; school engagement with parents; and school financial considerations. In addition, socioeconomic and demographic characteristics of the pupil population will be assessed through routinely available school data. Information on SFS voluntary sign up will also be requested from non-mandated schools.

##### Data capture on SFS/SFP resource use

Food provision costs will be estimated from a detailed list of resources obtained through a school staff survey, 1-day researcher observation, catering contracts, and school menus and pricing. Wider costs associated with SFP implementation will be obtained from school staff, parent and pupil surveys and observation. Each resource use item will be costed using financial data supplied by the school or obtained from the published literature.

##### Pupil outcome data collection

We aim to compare dietary intake and dental health in pupils in the two defined SFS school groups. The primary outcomes are intake of free sugars (grams): (1) during school day lunch (determined by asking pupils when they ate their lunch); (2) while at school; and (iii) during the full 24-hour period of the same school day. Secondary dietary outcomes include: percentage of dietary energy intake from free sugars; total energy intake (kcal); total fat intake (grams); fibre intake (grams); number of sugar-sweetened beverages consumed; number of sugar and chocolate confectionery items consumed; number of foods high in fat, sugar and salt consumed; and number of fruit and vegetable portions consumed. These outcomes will also be compared across the two school groups for the three defined time periods (school day lunch; while in school; over 24 hours). Additional secondary dietary outcomes are: free sugar intake providing >5% energy intake; number of eating/drinking occasions (excluding plain water); and consumption of five or more portions of fruit and vegetables per day. Secondary outcomes relating to dental health are: the presence of dental caries; the number of dental caries symptoms; and treatment received for dental caries.

Self-reported pupil data will be collected in school, during two timetabled sessions approximately 1–4 weeks apart. Pupils will complete an online survey at each session followed by a 24-hour recall dietary assessment. The first and second sessions will be facilitated by trained researchers and classroom teachers, respectively. An alternative activity will be provided for non-participating pupils.

###### Dietary intake assessment

Dietary intake will be measured using Intake24, an online self-completion 24-hour dietary recall tool based on the multiple pass method, which has been shown to be the most accurate for assessing adolescent dietary intake.^27^ Compared with interviewer-led recall, Intake24 underestimated energy intake by just 1% in this age group, and differences in mean macronutrient/micronutrient intakes between the two methods were within 4%.^28^

Nutrient analysis of Intake24 data uses the National Diet and Nutrition Survey food database containing over 2300 foods linked to the UK Nutrient Databank codes.^29^ Intake24 piloting, community nutritionist consultation and literature review identified food/drinks commonly consumed by minority ethnic groups but not included in this database (n=63), which were added to Intake24. Nutritional data for these items were obtained through matching to existing items in Intake24 or from other existing food composition sources.^30 31^ Photographs are used for portion size estimation, a method which has shown good agreement with 4-day weighed intakes in adolescents.^32 33^

###### Assessment of dental caries experience

We will use validated self-report measures from the national Child Dental Health Survey^2^ to assess dental caries symptoms in the last 3 months and treatment received in the last 24 months. Self-reported tooth brushing frequency data will also be collected.

###### Other pupil data collection

Pupils will be asked to provide their age, sex, ethnicity, postcode (for mapping to Index of Multiple Deprivation (IMD) scores and exposure to fluoridated water), usual lunch type (school-provided vs home-packed), usual mode of travel to/from school and sleep/wake times for the previous night. Pupils will also complete the Child Health Utilities 9-Dimension (CHU-9D) questionnaire, a utility-based health-related quality of life questionnaire,^34^ for use within the economic evaluation.

##### Data collection tool piloting

The school staff, governor, pupil and parent surveys, the adapted Intake24 and the observation tool were piloted with the relevant target groups. The tools were revised in line with the feedback obtained.

#### Data analysis

##### Preliminary development of a school typology

Using the logic model (figure 1) as a guide, we will use data captured on SFS/SFP implementation and the school context to develop an initial typology of schools, which will reflect the degree of implementation of the SFS/SFP and other relevant local initiatives relating to food and healthy eating.

##### Analysis of pupil outcomes

Pupils will have a maximum of two 24-hour recalls for dietary intake measures. To summarise dietary outcomes, we will report the average value across the 2 days for each pupil. If only a single day is available, we will use data from that day only. Differences between the SFS mandated and SFS non-mandated school groups for both primary and secondary pupil outcomes will be assessed using multilevel linear models that account for clustering at the school and class level (and at the Multi-Academy Trust level if applicable). Models will include adjustment for propensity scores and pupil-level covariates (school year group, sex, ethnicity, IMD score and school-provided vs home-packed lunch). Models for dietary outcomes will use data recorded each day, allowing for the repeated outcome measures. Models for dental outcomes will be additionally adjusted for oral healthcare factors and water fluoridation exposure. We will also explore whether the associations between school SFS status and pupil outcomes differ across age, socioeconomic position (indicated by IMD score), or usual lunch type (school-provided vs home-packed) by adding the relevant interaction terms to the developed models. If more than 5% of data are missing from demographic variables, multiple imputation methods will be used and sensitivity analyses conducted.

If possible, within the group of schools which are not mandated to adhere to the SFS, we will undertake an exploratory analysis to examine differences in pupil outcomes between schools who have and have not voluntarily signed up to the SFS. In addition, we will develop further linear multilevel models to explore potential associations between the identified school types and pupil outcomes, again adjusting for clustering, potential pupil-level confounders and propensity scores, as described previously.

### Phase II: school case studies

A number of schools (n=4–8) will be asked to participate in a qualitative study. Schools will be selected to comprise a range of school types and include at least two schools from the SFS non-mandated group not reporting voluntary sign up. These schools will receive a further £150.

We will undertake interviews with key school stakeholders (identified by the school Senior Leadership Team, aiming for 4–6 per school) to explore in more depth the way in which the SFS/SFP and local school policy or initiatives are introduced, embedded and sustained in the schools, and their perceived influence on the dietary intake of pupils. Data collection will be shaped with reference to the four constructs, and their constituent components, within May *et al*’s NPT^25^ and by Maguire *et al*’s exploration of policy enactment in English schools.^35^ Interviewees will be encouraged to tell their ‘story’ relating to their experiences of the SFS/SFP; how the provision of food, eating environments and the food/cooking curriculum have been shaped within their schools; and the influence of the wider school context.

Through focus groups (FGs) with school pupils (aiming for one FG in each included year group per school, comprising 6–8 participants per FG) we will explore their views of the school food environment, contextual factors influencing this, and how they interact with this and the wider external environment surrounding the school, in terms of their eating behaviour. We will also explore the views and experiences of any negative impact of SFS/SFP and the regulation of foods provided. Participating pupils will receive a £5 voucher.

Interviews/FGs will be audio-recorded with participant consent, transcribed ad verbatim and anonymised. We will use the framework analytical approach,^36^ and within this undertake thematic analysis, guided by NPT and Maguire *et al*’s policy enactment exploration.^25 35^

### Economic evaluation

To evaluate the economic impact of the SFS and the wider SFP, a cost–consequence analysis will be undertaken. We will summarise costs and outcomes in the form of a balance sheet. The analysis will highlight the costs to schools and families, and offer a transparent range of outcome measures for consideration. In addition, we will conduct an exploratory cost–utility analysis. Quality-Adjusted Life Years (QALYs) will be constructed from pupil responses to CHU-9D to allow inferences to be made about QALY-differences between the two school groups offset against the cost.

### Patient and public involvement

During research plan development, the deputy headteacher member of the investigator team advised on engaging schools and pupils and access to food sales data and provided background information on school management and governance systems. In addition, we consulted with a group of parents of secondary school children and a group of secondary school pupils, a teacher and a manager of a school catering company, who advised on school, pupil and parent recruitment, data collection methods and survey content. To provide ongoing advice from a public perspective, we have convened a group of parents, teachers and secondary school governors (n=8), and two groups of secondary school pupils to consult with at key points throughout the study. To date, these groups have reviewed and piloted surveys and Intake24, and advised on developing a safeguarding policy; school, staff, pupil and parent recruitment and school retention; data collection practicalities; and the alternative activity for non-participating pupils.

## Ethics and dissemination

Full ethical approval for the study was obtained from the University of Birmingham Science, Technology, Engineering and Mathematics Ethical Review Committee on 20 August 2019 (ERN_18-1738).

### Data management and study oversight

The University of Birmingham is the Study Sponsor and data controller and assumes overall responsibility for the study. Data management and storage is compliant with the UK Data Protection Act 2018 and follows the relevant University of Birmingham policy and procedures. Anonymised data will be stored securely for a minimum of 10 years after the publication of the main study results.

An independently chaired Study Steering Committee (SSC) has been convened to provide study oversight. Membership comprises three independent academics with relevant expertise, a representative from Public Health England (PHE), a public representative and the Chief Investigator. The Committee has agreed on the current protocol and will review subsequent amendments.

### Dissemination

Study findings will be disseminated to key national agencies (eg, Department of Health and Social Care, DfE, PHE, School Food Alliance, National Association for Headteachers and the Lead Association for Caterers in Education (LACA)), local level organisations (eg, Regional School Commissioners and Local Authorities), and schools through reports, conference/meeting presentations, the educational and general press and open access publications. The public representatives on the Investigator team, SSC and other public engagement groups will advise further on dissemination plans for the study findings. A full report of the study will be published in the *NIHR Journals Library*. After the publication of the main study findings, anonymised data will be available on request from the study Chief Investigators.

## Supplementary Material

Reviewer comments

Author's manuscript
