# Supplementary figures and images for: The Food provision, cUlture and Environment in secondary schooLs (FUEL) study: protocol of a mixed methods evaluation of national School Food Standards implementation in secondary schools and their impact on pupils’ dietary intake and dental health
Source: BMJ Open. 2020 Oct 16;10(10):e042931. doi: 10.1136/bmjopen-2020-042931 (PMC7569925; doi:10.1136/bmjopen-2020-042931)

## Recruitment and data collection flowchart

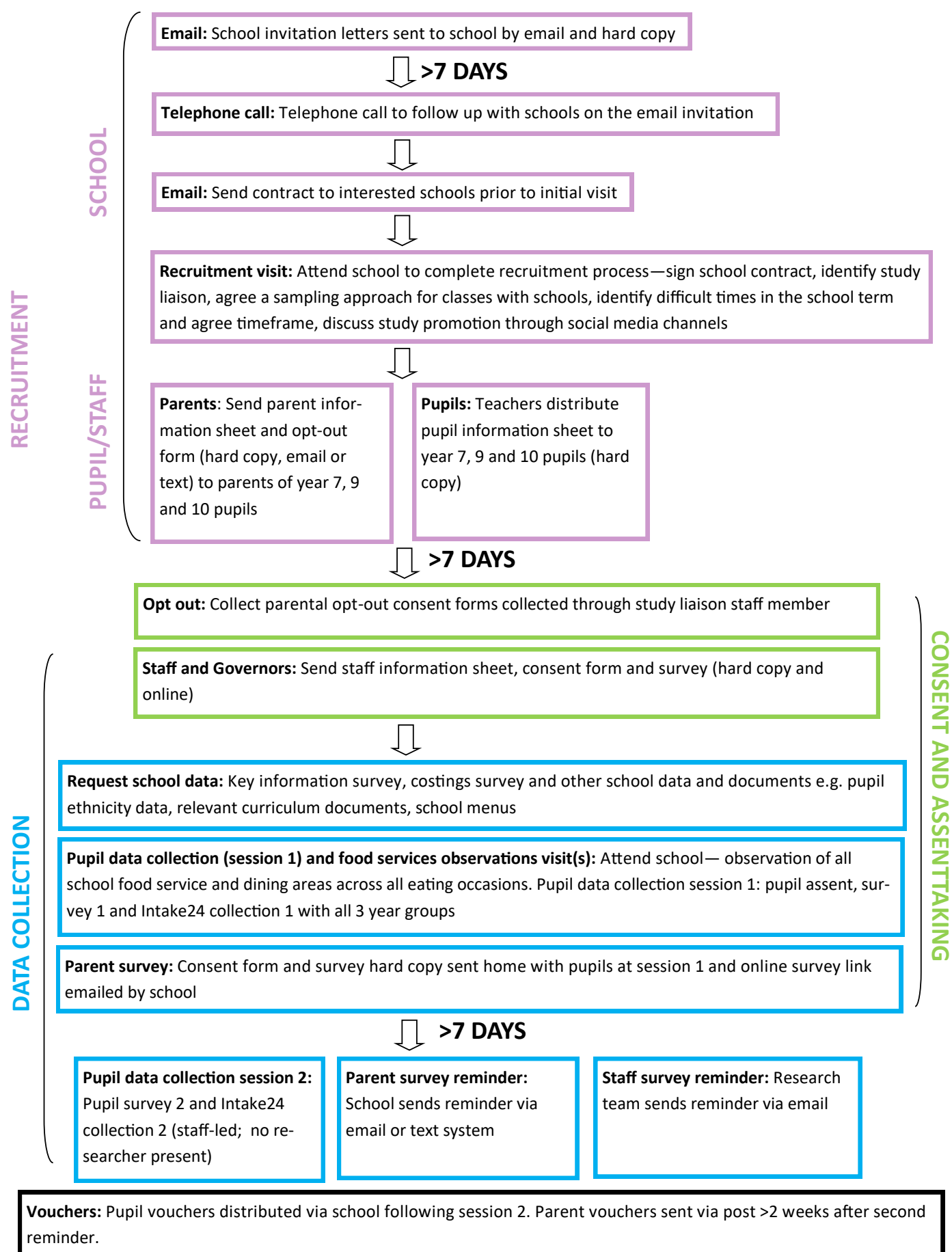

Supplement: Supplementary data [file bmjopen-2020-042931supp001.pdf]
