# Supplementary material for: The Food provision, cUlture and Environment in secondary schooLs (FUEL) study: protocol of a mixed methods evaluation of national School Food Standards implementation in secondary schools and their impact on pupils’ dietary intake and dental health
Source: BMJ Open. 2020 Oct 16;10(10):e042931. doi: 10.1136/bmjopen-2020-042931 (PMC7569925; doi:10.1136/bmjopen-2020-042931)
Supplement: Author's manuscript [file bmjopen-2020-042931.draft_revisions.pdf]

BMJ Open

BMJ Open is committed to open peer review. As part of this commitment we make the peer review history of every article we publish publicly available.

When an article is published we post the peer reviewers' comments and the authors' responses online. We also post the versions of the paper that were used during peer review. These are the versions that the peer review comments apply to.

The versions of the paper that follow are the versions that were submitted during the peer review process. They are not the versions of record or the final published versions. They should not be cited or distributed as the published version of this manuscript.

BMJ Open is an open access journal and the full, final, typeset and author-corrected version of record of the manuscript is available on our site with no access controls, subscription charges or pay-per-view fees (http://bmjopen.bmj.com).

If you have any questions on BMJ Open's open peer review process please email info.bmjopen@bmj.com

BMJ Open

The Food provision, cUlture and Environment in secondary schooLs (FUEL) study: protocol of a mixed methods evaluation of national School Food Standards implementation in secondary schools and their impact on pupils' dietary intake and dental health

Journal:	*BMJ Open*
Manuscript ID	bmjopen-2020-042931
Article Type:	Protocol
Date Submitted by the Author:	19-Jul-2020
Complete List of Authors:	Murphy, Marie; University of Birmingham, Institute of Applied Health Research<br>Pallan, Miranda; University of Birmingham, Institute of Applied Health Research<br>Lancashire, Emma; University of Birmingham, Institute of Applied Health Research<br>Duff, Rhona; University of Birmingham, Institute of Applied Health Research<br>Adamson, Ashley; University of Newcastle, Human Nutrition Research CentreInstitute of Health & Society<br>Bartington, Suzanne; University of Birmingham, Institute of Applied Health Research<br>Frew, Emma; University of Birmingham, Institute of Applied Health Research<br>Griffin, Tania ; University of Bath,<br>Hurley, Kiya; University of Birmingham, Cancer Research UK Clinical Trials Unit<br>Parry, Jayne; University of Birmingham, Institute of Applied Health Research<br>Passmore, S; Services for Education Ltd,<br>Ravaghi, Vahid; University of Birmingham, School of Dentistry<br>Sitch, Alice; University of Birmingham, Institute of Applied Health Research; University Hospitals Birmingham NHS Foundation Trust, NIHR Birmingham Biomedical Research Centre<br>Spence, Suzanne; Newcastle University, Institute of Health & Society, Human Nutrition Research Centre<br>Rowland, Maisie; Newcastle University, Human Nutrition Research Centre and Population Health Sciences Institute<br>Wheeldon, Scott; Wheelers Lane Technology College<br>Adab, Peymane; University of Birmingham, Institute of Applied Health Research
Keywords:	EPIDEMIOLOGY, PUBLIC HEALTH, NUTRITION & DIETETICS

SCHOLARONE™
Manuscripts

I, the Submitting Author has the right to grant and does grant on behalf of all authors of the Work (as defined in the below author licence), an exclusive licence and/or a non-exclusive licence for contributions from authors who are: i) UK Crown employees; ii) where BMJ has agreed a CC-BY licence shall apply, and/or iii) in accordance with the terms applicable for US Federal Government officers or employees acting as part of their official duties; on a worldwide, perpetual, irrevocable, royalty-free basis to BMJ Publishing Group Ltd ("BMJ") its licensees and where the relevant Journal is co-owned by BMJ to the co-owners of the Journal, to publish the Work in this journal and any other BMJ products and to exploit all rights, as set out in our licence.

The Submitting Author accepts and understands that any supply made under these terms is made by BMJ to the Submitting Author unless you are acting as an employee on behalf of your employer or a postgraduate student of an affiliated institution which is paying any applicable article publishing charge ("APC") for Open Access articles. Where the Submitting Author wishes to make the Work available on an Open Access basis (and intends to pay the relevant APC), the terms of reuse of such Open Access shall be governed by a Creative Commons licence – details of these licences and which Creative Commons licence will apply to this Work are set out in our licence referred to above.

Other than as permitted in any relevant BMJ Author's Self Archiving Policies, I confirm this Work has not been accepted for publication elsewhere, is not being considered for publication elsewhere and does not duplicate material already published. I confirm all authors consent to publication of this Work and authorise the granting of this licence.

For peer review only

TITLE

The Food provision, cUlture and Environment in secondary schooLs (FUEL) study: protocol of a mixed methods evaluation of national School Food Standards implementation in secondary schools and their impact on pupils' dietary intake and dental health

AUTHORS

Marie Murphy[1] and Miranda Pallan[1] (joint first), Emma Lancashire[1], Rhona Duff[1], Ashley Adamson[2], Suzanne Bartington[1], Emma Frew[1], Tania Griffin[3], Kiya Hurley[4], Jayne Parry[1], Sandra Passmore[5], Vahid Ravaghi[6], Alice Sitch[1,7], Suzanne Spence[2], Maisie K Rowland[2], Scott Wheeldon[8], Peymane Adab[1]

1. Institute of Applied Health Research, University of Birmingham, Birmingham, UK
2. Human Nutrition Research Centre, Population Health Sciences Institute, Newcastle University, Newcastle, UK
3. Department for Health, University of Bath, Bath, UK
4. Cancer Clinical Trials Unit, University of Birmingham, Birmingham, UK
5. Services for Education, Birmingham, UK
6. School of Dentistry, University of Birmingham, Birmingham, UK
7. NIHR Birmingham Biomedical Research Centre, University Hospitals Birmingham NHS Foundation Trust and University of Birmingham, UK
8. Wheelers Lane Technology College, Birmingham, UK

Corresponding author

Miranda Pallan

Room 107, Murray Learning Centre

University of Birmingham

Edgbaston

Birmingham

UK

B15 2TT

Email: m.j.pallan@bham.ac.uk

Word count = 3,776 (excluding title page, abstract, contributions, acknowledgements, references, figures and tables)

For peer review only - http://bmjopen.bmj.com/site/about/guidelines.xhtml

KEY WORDS:

Food policy

Schools

Adolescents

Dietary intake

Dental health

ABBREVIATIONS

BAME = Black, Asian and Minority Ethnic

CHU-9D = Child Health Utility 9 Dimension questionnaire

DfE = Department for Education

EAL = English As a foreign Language

FG = Focus Group

FSM = Free School Meals

ICC = Intraclass-cluster Correlation Coefficient

IDACI = Income Deprivation Affecting Children Index

IMD = Index of Multiple Deprivation

ISRCTN = International Standard Randomised Controlled Trials Number

MAT = Multi-Academy Trust

NDNS = National Diet and Nutrition Survey

NPT = Normalization Process Theory

QALY = Quality Adjusted Life Year

SD = Standard Deviation

SEN = Special educational needs

SFS = School Food Standards

SFP = School Food Plan

SSC = Study Steering Committee

UK = United Kingdom

REGISTRATION

ISRCTN: 68757496 (registered 17th October 2019)

Protocol version 5.0 (1st May 2020)

For peer review only - http://bmjopen.bmj.com/site/about/guidelines.xhtml

ABSTRACT

Introduction: Excess free sugar intake is associated with obesity and poor dental health. Adolescents consume substantially more free sugar than is recommended. National (UK) School Food Standards (SFS) are in place, but are not mandatory in all schools, and their impact on the diets of secondary school pupils is unknown. We aim to evaluate how SFS and wider healthy eating recommendations (from the national School Food Plan (SFP)) are implemented in secondary schools, and how they influence pupils' diets and dental health.

Methods and analysis: Secondary-level academies/free schools in the West Midlands, UK were divided into two groups: SFS mandated; SFS non-mandated. Using propensity scores to guide sampling, we aim to recruit 22 schools in each group. We will compare data on school food provision and sales, school food culture and environment, and the food curriculum from each group, collected through: school staff, governor, pupil, parent surveys; school documents; and observation. We will explore implementation level for the SFS requirements and SFP recommendations, and develop a school food typology. We aim to recruit 1980 pupils aged 11-15 years across the 44 schools and collect dietary intake (24 hour recall) and dental health data through self-completion surveys. We will compare free sugar/other dietary intake and dental health across the two SFS groups, and across the identified school types. School type will be further characterised in 4-8 case study schools through school staff interviews and pupil focus groups. Evaluation of economic impact will be through a cost-consequence analysis and an exploratory cost-utility analysis.

Ethics and dissemination: Ethical approval was obtained from the University of Birmingham Ethical Review Committee (ERN_18-1738). Findings will be disseminated to key national and local agencies, schools and the public through reports, presentations, the media and open access publications.

Registration details: ISRCTN 68757496 (registered 17/10/2019)

For peer review only

For peer review only - http://bmjopen.bmj.com/site/about/guidelines.xhtml

ARTICLE SUMMARY

Strengths and limitations of this study

1. This research fills a gap in the literature by evaluating the impact of national school food policy upon the dietary intake of secondary school pupils in the UK, comparing schools that are mandated to adhere to the national School Food Standards to those that are not.
2. A validated online dietary assessment tool, adapted for use in an ethnically diverse population, will be used to collect dietary intake data from 1980 secondary school pupils across 44 schools in the West Midlands.
3. The research includes a comprehensive assessment of variation in the implementation of the School Food Standards, School Food Plan recommendations and other school contextual factors across schools through researcher observation, menu analysis, school document review, and surveys with school staff, governors, pupils and parents.
4. The study design includes a qualitative research element in a number of case study schools to provide an in-depth understanding of school food provision, environments and the culture/ethos relating to this.
5. This study is observational and the two groups of participating schools (mandated and non-mandated to adhere to the School Food Standards) may differ from one another – however, to mitigate this we have used stratified sampling based on propensity scores (estimated from a large number of school characteristics) to enable more comparability between the two school groups.

For peer review only

For peer review only - http://bmjopen.bmj.com/site/about/guidelines.xhtml

MAIN TEXT

INTRODUCTION

Excess sugar consumption is a major contributor to increased energy intake/obesity, adverse cardio-metabolic health,[1] and poor dental health.[2] UK adolescents consume three times their recommended amount of total energy intake from free sugars;[3] almost half of 15 year olds have dental caries;[2] and nearly a third have excess weight.[4] Adolescence is an important time for dietary intervention as it is a key period for establishing dietary patterns,[5] with greater autonomy over dietary decisions. A large proportion of adolescent dietary intake occurs whilst at school, making these opportune settings for intervention.

In the UK, a longstanding strategy for improving children's diet has been nutritional standards for school food. School meal standards were first introduced in 1941, but fell out of favour later in the 20th century. In 2006, following a national school meals review,[6] national School Food Standards (SFS) were relaunched in England and became a legal requirement for most state schools. In 2015, a Department for Education (DfE) SFS review resulted in substantial changes. For ease of implementation, nutrient-based standards were removed whilst retaining food-based standards underpinned by a nutrient framework.[7]

In addition to the national SFS, the School Food Plan (SFP) was launched in 2013, providing a wider set of non-statutory recommendations for schools that promote a 'whole school' approach to healthy eating.[8] One central aim was to increase school meal uptake, as higher demand enables better quality meals to be served at lower cost. Another aim was to provide practical support, advice and information for headteachers to help improve quality and uptake of their school's food. In addition to school food provision, the plan addresses how healthy eating can be incorporated within all aspects of school life and the wider community.[9]

Evaluation of the impact of the 2006 SFS on food provision and consumption has been conducted in primary (age 4-11 years) and middle (age 9-12 years) schools in the UK. Pre- and post- SFS implementation comparisons showed improvements in overall dietary intake in pupils aged 4-7 years, but not in those aged 11-12 years.[10-12] SFS implementation was good in primary schools but less so in middle schools.[10] Evidence of SFS impact on secondary school pupils' (aged 11-16 years) dietary intake is more limited. A study in 80 secondary schools compared school food provision in 2011 with that in 2004. They reported improvements in nutritional content of school-provided food (in particular reduced

For peer review only - http://bmjopen.bmj.com/site/about/guidelines.xhtml

confectionary availability) and pupils' lunchtime food consumption, but did not examine total dietary intake.[13]

To date, the impact of the updated SFS or the SFP has not been evaluated. The way in which SFS legislation was introduced in England means that certain school types (including academies and free schools; 70% of secondary schools in England)[14] which were set up between January 2010 and May 2014 are exempt from this legislation (although they can choose to voluntarily sign up to the standards), whereas schools of these types established before or after these dates are legally required to meet the SFS. This provides an opportunity to examine how a legal requirement to meet the SFS influences schools and their pupils. Given the lack of SFS /SFP evaluation in secondary schools, we aim to investigate their influence in these settings by comparing school food provision, sales, wider school factors related to food, pupil dietary intake and dental health across those mandated and not mandated to adhere to the current SFS. We will also explore variation in implementation of the SFS and SFP recommendations, and their economic impact.

METHODS AND ANALYSIS

This is an observational, mixed method design study, consisting of two phases. Phase 1 involves collecting a variety of data on SFS/SFP implementation, and school and pupil outcomes in the SFS mandated and non-mandated schools. We will compare outcomes across the two groups and develop a typology of schools based on SFS/SFP implementation and wider school contextual factors. In phase 2, we will identify a small number of 'case study' schools and conduct a qualitative inquiry to further develop the school typology. Furthermore, we will undertake an economic evaluation to assess how the costs and outcomes vary by school typology.

Study setting

The sampling frame comprises secondary phase academies/free schools providing education to children aged 11-16 years and located within 14 Local Authority areas in the West Midlands region, UK. This region has a population of five million (21% of ethnicity other than White British),[15] urban, suburban and rural areas, and areas of high socioeconomic deprivation.[16] Other school types and academies that provide specialist alternative education have been excluded.

Phase 1

For peer review only - http://bmjopen.bmj.com/site/about/guidelines.xhtml

Sampling

To increase comparability of the two school groups, stratified sampling, based on propensity scores, was used.[17] We obtained routine data from the Department for Education on several characteristics for all schools in the sampling frame: Local Authority area; establishment type; urban/rural; total pupil roll size; Income Deprivation Affecting Children Index (IDACI); inclusion of a sixth form; selective/non-selective admissions policy; religious affiliation/secular; and proportion of: male/female pupils; pupils from Black, Asian and minority ethnic groups (BAME); students with English as a foreign language (EAL); students eligible for free school meals (FSM); and pupils with Special Educational Needs (SEN). We developed propensity scores using linear regression with the SFS status of the school (mandated/non-mandated) as the outcome and school characteristics as explanatory variables. Propensity score quartiles were then used to create four groups with subsequent division by SFS status (based on the date they received academy/free school status), resulting in eight distinct sampling groups. Following random ordering of each group, schools will be invited sequentially to participate, aiming to recruit five or six schools from each group.

Within participating schools, pupils in one class from each of years 7, 9 and 10 (aged 11-12, 13-14 and 14-15 years, respectively) will be invited to take part. Preference will be given to classes not streamed by academic ability or subject to enable participation of classes which are representative of the year group characteristics. There are no pupil exclusion criteria.

Sample size calculation

We used data on free sugar intake pre- and post-SFS implementation from the study undertaken in middle schools by Adamson et al.[10] to inform our sample size calculation. Assuming an intra-class correlation coefficient (ICC) of 0.1 [18] and balanced cluster sizes, we estimated that to detect a difference in mean free sugar intake at lunch of 4g (20g vs.16g) between the two school groups, assuming a SD of 11 with 90% power and at 5% significance, we require 990 evaluable participants and 22 clusters (conservatively using schools[19]) in each group (total schools=44; total participants n=1980; cluster size=45).

Recruitment

Headteachers will be invited by post and email with telephone follow-up. In participating schools a liaison staff member will be identified and a contract, outlining expected commitments from both parties, will be signed. Once data collection is complete, participating schools will receive a school-specific summary report and £300.

For peer review only - http://bmjopen.bmj.com/site/about/guidelines.xhtml

At least seven days prior to pupil data collection, pupils from the three selected classes will receive a participant information pack (comprising pupil and parent information sheets and a parental opt-out consent form). Schools will also be asked to e-mail documents directly to parents. Pupils whose parents do not return a completed opt-out consent form will be invited to take part in the data collection sessions during the school day and asked for their electronic assent for study participation. Parents of pupils in selected classes will be invited to complete a self-administered parent survey (online or paper copy). Both pupils and parents who participate receive a £5 voucher.

Participating schools will be asked to identify key staff/governors with roles relating to food provision, eating environment, food curriculum, or SFS/SFP implementation. Identified staff will be sent a staff participant information sheet (email/paper copy) and study invitation for a self-administered survey (online or paper format). Recruitment and data collection processes are outlined in Supplementary File 1.

School and pupil recruitment commenced in October and November 2019 respectively, and was due to be completed in the 2019/20 academic year. However, due to restrictions in place in England during the COVID-19 pandemic, recruitment and data collection was suspended in March 2020 and will recommence when schools re-open for all pupils to attend. All further data collection activities will be undertaken in accordance with relevant Department of Health and Social Care and Department for Education health protection guidance.

Data collection

Data capture on SFS and SFP implementation, and the wider school context

A logic model setting out the processes by which the SFS/SFP are assumed to generate pupil health gains has been developed (Figure 1). Briefly, we hypothesise health gain materialises directly via a change in school food consumption, and indirectly by curricular and other activities designed to change pupils' dietary knowledge, attitudes and beliefs which impact on food consumption in and out of school. The extent to which the SFS/SFP achieve these health gains depends on their implementation within a school, which is influenced by key contextual factors (e.g. school management, parental engagement etc.).

Our data collection strategy, guided by the principles for process evaluation outlined in the UK Medical Research Council guidance,[20] will provide data to populate and refine the logic model. This will enable us to assess the extent to which the SFS requirements and SFP

For peer review only - http://bmjopen.bmj.com/site/about/guidelines.xhtml

recommendations are embedded in each school, and the relative importance of contextual factors in influencing this. Our data collection methods include: school menu analysis; observation of school food provision and the related environment; school document review; collection of aggregated routine data on pupil characteristics; and self-administered surveys for staff, governors, pupils and parents.

Assessment of SFS compliance and SFP recommendations implementation

SFS compliance assessment is based on the national SFS checklists for school lunch and school food other than lunch [21] that consist of daily, weekly and three-weekly criteria for food and drink offered. Using an observation tool developed to measure compliance with the SFS daily criteria, trained researchers will record food and drink provision at all eating occasions across one school day, and at all school food outlets and dining areas. Compliance with the SFS weekly and three-weekly criteria will be assessed from schools' weekly menus.

Implementation of the SFP recommendations is based on three guidance documents produced by the DfE: 1) a headteacher checklist; 2) a guide to creating a culture and ethos of healthy eating; and 3) guidance for governors.[22-24] Implementation will be assessed through questions in the surveys to pupils, parents, staff and governors; researcher observation of food outlets and dining areas; and school document review, e.g. school food policy, catering contract, curriculum documents and minutes of the Board of Governors and School Council meetings.

The school staff and governor surveys include a series of questions based on Normalization Process Theory (NPT),[25] to explore the implementation and sustained embedding of the SFS/SFP. The items in the NoMAD instrument (developed to assess the four NPT constructs),[26] were adapted for relevance to the SFS/SFP, and the school setting.

Assessment of school lunch uptake and food sales

To assess school lunch uptake, schools will be asked to provide their routinely collected school meal uptake data. To assess school food sales, we will request weekly aggregated food sales data for two pre-specified months in the previous year. Most schools use online payment management systems, which provide sales data by types of food/drinks sold. We will extract data on the number of items sold for both SFS restricted food/drinks (including sugar sweetened beverages, confectionary, fried food, snacks etc.) and pre-defined healthy foods and compare sales of these food categories across schools, taking into account school size and school lunch uptake.

For peer review only - http://bmjopen.bmj.com/site/about/guidelines.xhtml

For peer review only

Assessment of school contextual factors

We will collect data on a variety of contextual factors through observation, school document review, and the staff, governor, pupil and parent surveys, with further exploration in the second phase case studies. These factors include: the physical school environment; school leadership and organisational culture; the influence of governors; staff knowledge and skills; school engagement with parents; and school financial considerations. In addition, socioeconomic and demographic characteristics of the pupil population will be assessed through routinely available school data. Information on SFS voluntary sign-up will also be requested from non-mandated schools.

Data capture on SFS/SFP resource use

Food provision costs will be estimated from a detailed list of resources obtained through a school staff survey, one-day researcher observation, catering contracts, and school menus and pricing. Wider costs associated with SFP implementation will be obtained from school staff, parent and pupil surveys and observation. Each resource use item will be costed using financial data supplied by the school, or obtained from published literature.

Pupil outcome data collection

We aim to compare dietary intake and dental health in pupils in the two defined SFS school groups. The primary outcomes are intake of free sugars (grams): i) during school day lunch (determined by asking pupils when they ate their lunch); ii) whilst at school; and iii) during the full 24 hour period of the same school day. Secondary dietary outcomes include: percentage of dietary energy intake from free sugars; total energy intake (kcal); total fat intake (grams); fibre intake (grams); number of sugar-sweetened beverages consumed; number of sugar and chocolate confectionary items consumed; number of foods high in fat, sugar and salt consumed; and number of fruit and vegetable portions consumed. These outcomes will also be compared across the two school groups for the three defined time-periods (school day lunch; whilst in school; over 24 hours). Additional secondary dietary outcomes are: free sugar intake providing >5% energy intake; number of eating/drinking occasions (excluding plain water); and consumption of five or more portions of fruit and vegetables per day. Secondary outcomes relating to dental health are: the presence of dental caries; the number of dental caries symptoms; and treatment received for dental caries.

Self-reported pupil data will be collected in school, during two timetabled sessions approximately 1-4 weeks apart. Pupils will complete an online survey at each session followed by a 24 hour recall dietary assessment. The first and second sessions will be facilitated by

For peer review only - http://bmjopen.bmj.com/site/about/guidelines.xhtml

For peer review only

trained researchers and classroom teachers, respectively. An alternative activity will be provided for non-participating pupils.

Dietary intake assessment:

Dietary intake will be measured using Intake24, an online self-completion 24 hour dietary recall tool based on the multiple pass method, which has been shown to be the most accurate for assessing adolescent dietary intake.[27] Compared with interviewer-led recall, Intake24 under-estimated energy intake by just 1% in this age group, and differences in mean macro-/micro-nutrient intakes between the two methods were within 4%.[28]

Nutrient analysis of Intake24 data uses the National Diet and Nutrition Survey (NDNS) food database containing over 2300 foods linked to the UK Nutrient Databank codes.[29] Intake24 piloting, community nutritionist consultation and literature review identified food/drinks commonly consumed by minority ethnic groups but not included in this database (n=63), which were added to Intake24. Nutritional data for these items were obtained through matching to existing items in Intake24 or from other existing food composition sources.[30, 31] Photographs are used for portion size estimation, a method which has shown good agreement with four day weighed intakes in adolescents.[32, 33]

Assessment of dental caries experience:

We will use validated self-report measures from the national Child Dental Health Survey [2] to assess dental caries symptoms in the last 3 months and treatment received in the last 24 months. Self-reported tooth brushing frequency data will also be collected.

Other pupil data collection:

Pupils will be asked to provide their age, sex, ethnicity, postcode (for mapping to Index of Multiple Deprivation (IMD) scores and exposure to fluoridated water), usual lunch type (school-provided vs. home-packed), usual mode of travel to/from school, and sleep/wake times for the previous night. Pupils will also complete the Child-Health Utilities 9-Dimension (CHU-9D), a utility-based health-related quality of life questionnaire,[34] for use within the economic evaluation.

Data collection tool piloting

The school staff, governor, pupil and parent surveys, the adapted Intake24 and the observation tool were piloted with the relevant target groups. The tools were revised in line with the feedback obtained.

For peer review only - http://bmjopen.bmj.com/site/about/guidelines.xhtml

For peer review only

Data analysis

Preliminary development of a school typology

Using the logic model (Figure 1) as a guide, we will use data captured on SFS/SFP implementation and the school context to develop an initial typology of schools, which will reflect the degree of implementation of the SFS/SFP and other relevant local initiatives relating to food and healthy eating.

Analysis of pupil outcomes

Pupils will have a maximum of two 24 hour recalls for dietary intake measures. To summarise dietary outcomes, we will report the average value across the two days for each pupil. If only a single day is available, we will use data from that day only. Differences between the SFS mandated and SFS non-mandated school groups for both primary and secondary pupil outcomes will be assessed using multilevel linear models that account for clustering at the school and class level (and at the Multi-Academy Trust level if applicable). Models will include adjustment for propensity scores and pupil-level covariates (school year group, sex, ethnicity, IMD score and school-provided vs home-packed lunch). Models for dietary outcomes will use data recorded each day, allowing for the repeated outcome measures. Models for dental outcomes will be additionally adjusted for oral healthcare factors and water fluoridation exposure. We will also explore whether the associations between school SFS status and pupil outcomes differ across age, socioeconomic position (indicated by IMD score), or usual lunch type (school-provided vs. home-packed) by adding the relevant interaction terms to the developed models. If more than 5% of data are missing from demographic variables, multiple imputation methods will be used and sensitivity analyses conducted.

If possible, within the group of schools not-mandated to adhere to the SFS, we will undertake an exploratory analysis to examine differences in pupil outcomes between schools who have and have not voluntarily signed up to the SFS. In addition, we will develop further linear multilevel models to explore potential associations between the identified school types and pupil outcomes, again adjusting for clustering, potential pupil-level confounders and propensity scores, as described above.

Phase 2: school case studies

A number of schools (n=4-8) will be asked to participate in a qualitative study. Schools will be selected to comprise a range of school types and include at least two schools from the SFS non-mandated group not reporting voluntary sign-up. These schools will receive a further £150.

For peer review only - http://bmjopen.bmj.com/site/about/guidelines.xhtml

We will undertake interviews with key school stakeholders (identified by the school Senior Leadership Team, aiming for 4-6 per school) to explore in more depth the way in which the SFS/SFP and local school policy or initiatives are introduced, embedded and sustained in the schools, and their perceived influence on the dietary intake of pupils. Data collection will be shaped with reference to the four constructs, and their constituent components, within May's NPT[25] and by Maguire's exploration of policy enactment in English schools.[35] Interviewees will be encouraged to tell their 'story' relating to their experiences of the SFS/SFP; how the provision of food, eating environments and the food/cooking curriculum have been shaped within their schools; and the influence of the wider school context.

Through focus groups (FGs) with school pupils (aiming for one FG in each included year group per school, comprising 6-8 participants per FG) we will explore their views of the school food environment, contextual factors influencing this, and how they interact with this and the wider external environment surrounding the school, in terms of their eating behaviour. We will also explore views and experiences of any negative impact of SFS/SFP and regulation of foods provided. Participating pupils will receive a £5 voucher.

Interviews/FGs will be audio-recorded with participant consent, transcribed ad verbatim and anonymised. We will use the framework analytical approach,[36] and within this undertake thematic analysis, guided by NPT and Maguire's policy enactment exploration.[25, 35]

Economic evaluation

To evaluate economic impact of the SFS and the wider SFP a cost-consequence analysis will be undertaken. We will summarise costs and outcomes in the form of a balance sheet. The analysis will highlight the costs to schools and families, and offer a transparent range of outcome measures for consideration. In addition, we will conduct an exploratory cost-utility analysis. Quality-Adjusted Life Years (QALYs) will be constructed from pupil responses to CHU-9D to allow inferences to be made about QALY-differences between the two school groups offset against cost.

Patient and public involvement

During research plan development, the deputy headteacher member of the investigator team advised on engaging schools and pupils and access to food sales data, and provided background information on school management and governance systems. In addition we consulted with a group of parents of secondary school children, a group of secondary school pupils, a teacher and

For peer review only - http://bmjopen.bmj.com/site/about/guidelines.xhtml

a manager of a school catering company, who advised on school, pupil and parent recruitment, data collection methods and survey content. To provide ongoing advice from a public perspective, we have convened a group of parents, teachers and secondary school governors (n=8), and two groups of secondary school pupils to consult with at key points throughout the study. To date these groups have reviewed and piloted surveys and Intake24, and advised on: developing a safeguarding policy; school, staff, pupil and parent recruitment and school retention; data collection practicalities; and the alternative activity for non-participating pupils.

ETHICS AND DISSEMINATION

Full ethical approval for the study was obtained from the University of Birmingham Science, Technology, Engineering and Mathematics Ethical Review Committee on 20th August 2019 (ERN_18-1738).

Data management and study oversight

The University of Birmingham is the Study Sponsor and data controller and assumes overall responsibility for the study. Data management and storage is compliant with the UK Data Protection Act 2018 and follows relevant University of Birmingham policy and procedures. Anonymised data will be stored securely for a minimum of 10 years after the publication of the main study results.

An independently chaired Study Steering Committee (SSC) has been convened to provide study oversight. Membership comprises three independent academics with relevant expertise, a representative from Public Health England (PHE), a public representative and the Chief Investigator. The Committee has agreed the current protocol and will review subsequent amendments.

Dissemination

Study findings will be disseminated to key national agencies (e.g. Department of Health and Social Care, DfE, PHE, School Food Alliance, National Association for Headteachers), local level organisations (e.g. Regional School Commissioners and Local Authorities), and schools through reports, conference/meeting presentations, the educational and general press and open access publications. The public representatives on the Investigator team, SSC and other public engagement groups will advise further on dissemination plans for the study findings. A full report of the study will be published in the NIHR Journals Library. After publication of the main study findings, anonymised data will be available on request from the study Chief Investigators.

For peer review only - http://bmjopen.bmj.com/site/about/guidelines.xhtml

AUTHOR CONTRIBUTIONS

MP/PA are the Chief Investigators with overall responsibility for the study. They conceived the study with input from SB and EL. MP and PA led on protocol development with contribution from all named authors. MP, PA, EL, MM, and RD developed the data collection tools. AA and MKR designed the dietary data collection tool Intake24, and RD, MKR, MM and MP contributed to the adaptation and piloting of Intake24 for the purposes of this study. AS designed and conducted the sampling procedure, and developed the analysis plan. EF designed the economic evaluation. VR advised on dental outcome measures. JP advised on the case study methodology. SP and SW advised on recruiting and working within schools. SS advised on the procurement and analysis of school food sales data. TG and KH advised on a range of methodological and practical aspects of the study. MM, MP, EL and RD drafted the manuscript and all authors critically reviewed the manuscript and agreed the final draft.

FUNDING STATEMENT

This study is funded by the National Institute for Health Research (NIHR) Public Health Research programme (17/92/39). Alice Sitch is part-funded by the NIHR Birmingham Biomedical Research Centre at the University Hospitals Birmingham NHS Foundation Trust. The views expressed are those of the authors and not necessarily those of the NIHR, NHS or the Department of Health and Social Care.

ACKNOWLEDGMENTS

Thank you to our Study Steering Group members Sheila Birdi, Helen Carter, Ann Hughes, Christopher Owen, Alexander Turner and Robert West (Chair) for their guidance. We are grateful for the contributions that our public advisors have made, including pupils and teachers at two schools in the West Midlands. We are also grateful to all those individuals who piloted study materials or provided advice on food/drink items commonly consumed by minority ethnic groups in the West Midlands.

COMPETING INTERESTS STATEMENT

Miranda Pallan, Emma Lancashire, Ashley Adamson, Suzanne Bartington, Emma Frew, Tania Griffin, Kiya Hurley, Jayne Parry, Sandra Passmore, Vahid Ravaghi, Alice Sitch and Peymane Adab hold grants from the UK National Institute for Health Research (NIHR). Peymane Adab is

For peer review only

Chair of the NIHR Public Health Research Funding Committee. Ashley Adamson is Director of the NIHR School for Public Health Research, a NIHR Senior Investigator and a member of both the School Food Plan Alliance and UK Prevention Research Partnership network Generating Excellent Nutrition in UK Schools (GENIUS). Jayne Parry is Chair of a NIHR Fellowships selection committee. Alice Sitch is a grant holder for the Birmingham NIHR Biomedical Research Centre. Suzanne Bartington is an elected member of Oxfordshire County Council. There are no other competing interests to declare.

REFERENCES

1. Johnson RK, Appel LJ, Brands M, et al. Dietary sugars intake and cardiovascular health: a scientific statement from the American Heart Association. *Circulation* 2009;120:1011-1020.
2. Holmes R, Porter J, Vernazza C, et al. Children's Dental Health Survey 2013. Country Specific Report: England, 2015.
3. Bates B, Cox L, Nicholson S, et al. Results of the National Diet and Nutrition Survey (NDNS) rolling programme for 2012 to 2013 and 2013 to 2014. London, 2016.
4. Conolly A: Health Survey for England 2015: Children's body mass index, overweight and obesity. Leeds, 2016.
5. Wang Y, Bentley ME, Zhai F, et al. Tracking of dietary intake patterns of Chinese from childhood to adolescence over a six-year follow-up period. *J Nutr* 2002;132:430-38.
6. Hope P. Statutory Instrument 2006 No. 2381 The Education (Nutritional Standards for School Lunches) (England) Regulations. London, 2006.
7. Mucavele P, Nicholas J, Sharp L. Development and pilot testing of revised food-based standards for school lunches in England. Sheffield: Children's Food Trust,; 2013. Available from: https://www.schoolfoodplan.com/wp-content/uploads/2014/02/School-Food-Plan-Pilot-study-EVALUATION-REPORT-Final-V3.pdf accessed June 2019.
8. The School Food Plan. The Independent School Food Plan 2019. Available from: http://www.schoolfoodplan.com/ accessed June 2019.
9. Dimbleby H, Vincent J. The School Food Plan 201. Available from: http://www.schoolfoodplan.com/wp-content/uploads/2013/07/School_Food_Plan_2013.pdf accessed May 2020.
10. Adamson AJ, White M, Stead M. The process and impact of change in the school food policy on food and nutrient intake of children aged 4-7 and 11-12 years both in and out of school: a mixed methods approach: Public Health Research Consortium 2012.
11. Spence S, Delve J, Stamp E, et al. The impact of food and nutrient-based standards on primary school children's lunch and total dietary intake: a natural experimental evaluation of government policy in England. *PloS ONE* 2013;8:e78298.
12. Spence S, Matthews JN, White M, et al. A repeat cross-sectional study examining the equitable impact of nutritional standards for school lunches in England in 2008 on the diets of 4-7y olds across the socio-economic spectrum. *Int J Behav Nutr Phys Act* 2014;11:128.

For peer review only - http://bmjopen.bmj.com/site/about/guidelines.xhtml

13. Nicholas J, Wood L, Harper C, et al. The impact of the food-based and nutrient-based standards on lunchtime food and drink provision and consumption in secondary schools in England. *Public Health Nutr* 2013;16:1052-65.

14. Morse A. Converting maintained schools to academies. London: National Audit Office,, 2018.

15. Gov.uk. Regional ethnic diversity 2018. Available from: https://www.ethnicity-facts-figures.service.gov.uk/uk-population-by-ethnicity/national-and-regional-populations/regional-ethnic-diversity/latest#areas-of-england-and-wales-by-ethnicity accessed April 2020.

16. Medland A. Portrait of the West Midlands. In: Office for National Statistics, ed., 2011.

17. Rubin DB. Estimating causal effects from large data sets using propensity scores. *Ann Intern Med* 1997;127:757-63.

18. Juras R. Estimates of Intraclass Correlation Coefficients and Other Design Parameters for Studies of School-Based Nutritional Interventions. *Eval Rev* 2016;40:314-33.

19. Hemming K, Lilford R, Girling AJ: Stepped-wedge cluster randomised controlled trials: a generic framework including parallel and multiple-level designs. *Stat Med* 2015;34:181-196.

20. Moore G, Audrey S, Barker M, et al. Process evaluation of complex interventions: a summary of Medical Research Council guidance. . In: Richards D, Hallberg IR, eds. Complex interventions in health: an overview of research methods. Abingdon: Routledge, 2015.

21. The School Food Plan. School food standards: a practical guide for schools their cooks and caterers 2016. Available from: http://www.schoolfoodplan.com/wp-content/uploads/2014/06/School-Food-Standards-Guidance-FINAL-V1a-140616.pdf accessed June 2019.

22. The School Food Plan. A checklist for head teachers 2013. Available from: https://www.schoolfoodmatters.org/sites/default/files/SFP-Headteacher-Checklist_0.pdf accessed June 2019.

23. The School Food Plan. Creating a Culture and Ethos of Healthy Eating 2013. Available from: http://www.schoolfoodplan.com/wp-content/uploads/2015/09/Creating-a-culture-ethos-of-healthy-eating-Practical-Guidance-Final.pdf accessed June 2019.

24. The School Food Plan. School Food: Guidance for Governors 2013. Available from: http://www.schoolfoodplan.com/wp-content/uploads/2016/03/School-Governors_Guidance.pdf accessed June 2019.

25. May C, Finch T. Implementing, Embedding, and Integrating Practices: An Outline of Normalization Process Theory. *Sociology* 2009;43:535-54.

26. Finch TL, Girling M, May CR, et al. NoMad: Implementation measure based on Normalization Process Theory. 2015. Available from: http://www.normalizationprocess.org accessed November 2019.

27. Burrows TL, Martin RJ, Collins CE. A systematic review of the validity of dietary assessment methods in children when compared with the method of doubly labeled water. *J Am Diet Assoc* 2010;110:1501-10.

28. Bradley J, Simpson E, Poliakov I, et al. Comparison of INTAKE24 (an Online 24-h Dietary Recall Tool) with Interviewer-Led 24-h Recall in 11-24 Year-Old. *Nutrients* 2016;8:358.

For peer review only - http://bmjopen.bmj.com/site/about/guidelines.xhtml

29. Roe M, Pinchen H, Church S, et al. McCance and Widdowson's the composition of foods seventh summary edition and updated composition of foods integrated dataset. *Nutr Bull* 2015;40:36-39.

30. Stadlmayr B, Charrondiere UR, Enujiugha VN, et al. West African Food Composition Table. Available from: http://www.fao.org/3/a-i2698b.pdf accessed August 2019.

31. Public Health England. Composition of foods integrated dataset (CoFID). Available from: https://www.gov.uk/government/publications/composition-of-foods-integrated-dataset-cofid accessed August 2019.

32. Foster E, Hawkins A, Barton KL, et al. Development of food photographs for use with children aged 18 months to 16 years: Comparison against weighed food diaries–The Young Person's Food Atlas (UK). *PloS ONE* 2017;12:e0169084.

33. Foster E, Matthews J, Lloyd J, et al. Children's estimates of food portion size: the development and evaluation of three portion size assessment tools for use with children. *Br J Nutr* 2008;99:175-84.

34. Stevens K. CHU-9D. Measuring health and calculating QALYs for children and adolescents. 2019. Available from: https://licensing.sheffield.ac.uk/i/health-outcomes/CHU-9D.html accessed December 2019.

35. Maguire M, Braun A, Ball S. 'Where you stand depends on where you sit': the social construction of policy enactments in the (English) secondary school. *Discourse: Studies in the Cultural Politics of Education* 2015;36:485-99.

36. Gale NK, Heath G, Cameron E, et al. Using the framework method for the analysis of qualitative data in multi-disciplinary health research. *BMC Med Res Methodol* 2013;13:117.

Figure 1: Logic model and theory of change for the influence of school food standards on children's dietary intake and health outcomes

Supplementary File 1: Recruitment and data collection flow chart

For peer review only - http://bmjopen.bmj.com/site/about/guidelines.xhtml

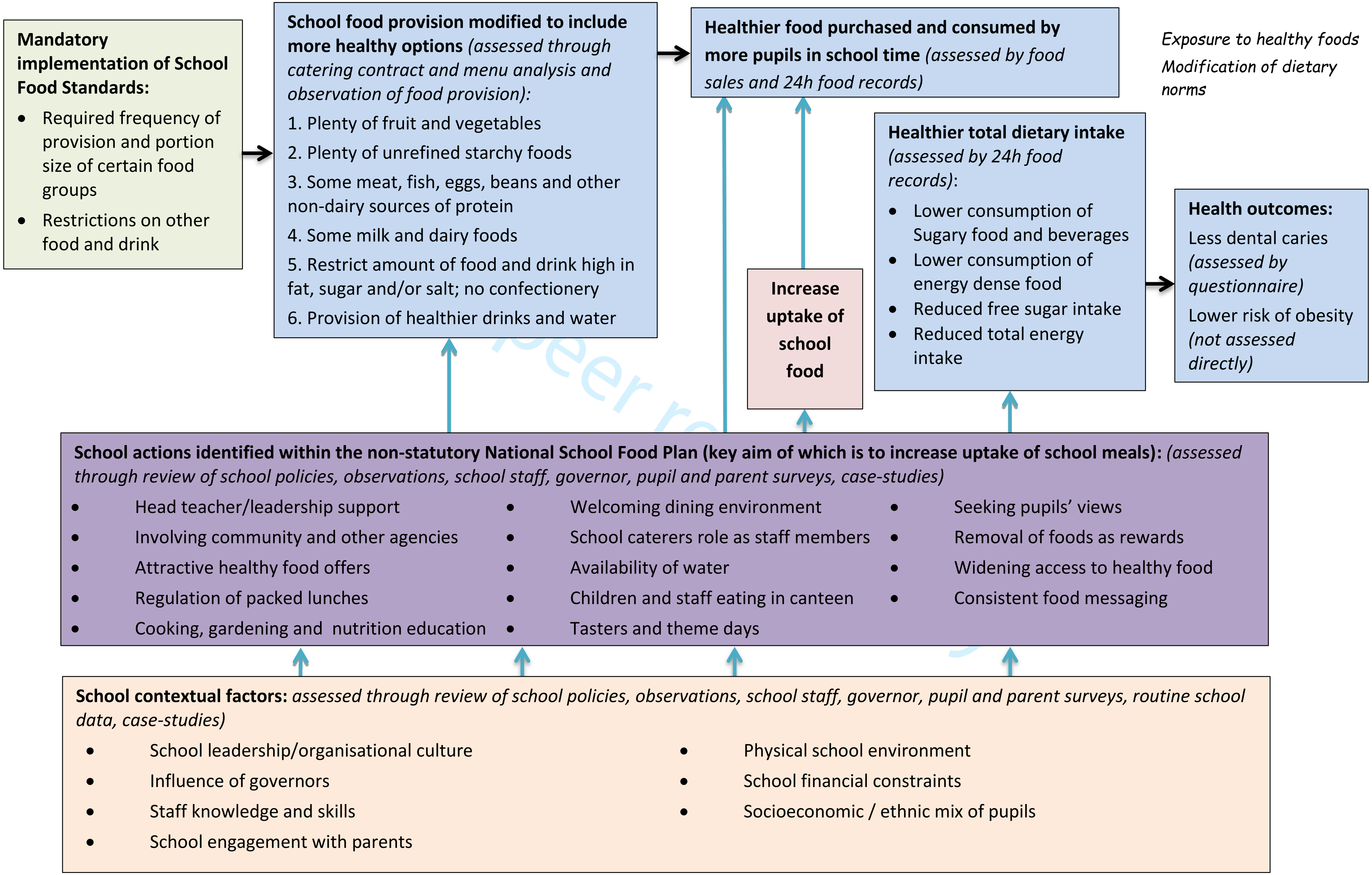
Mandatory implementation of School Food Standards:
Required frequency of provision and portion size of certain food groups
Restrictions on other food and drink
School food provision modified to include more healthy options (assessed through catering contract and menu analysis and observation of food provision):
1. Plenty of fruit and vegetables
2. Plenty of unrefined starchy foods
3. Some meat, fish, eggs, beans and other non-dairy sources of protein
4. Some milk and dairy foods
5. Restrict amount of food and drink high in fat, sugar and/or salt; no confectionery
6. Provision of healthier drinks and water
Healthier food purchased and consumed by more pupils in school time (assessed by food sales and 24h food records)
Exposure to healthy foods
Modification of dietary norms
Healthier total dietary intake (assessed by 24h food records):
Lower consumption of Sugary food and beverages
Lower consumption of energy dense food
Reduced free sugar intake
Reduced total energy intake
Health outcomes:
Less dental caries (assessed by questionnaire)
Lower risk of obesity (not assessed directly)
Increase uptake of school food
School actions identified within the non-statutory National School Food Plan (key aim of which is to increase uptake of school meals): (assessed through review of school policies, observations, school staff, governor, pupil and parent surveys, case-studies)
Head teacher/leadership support
Involving community and other agencies
Attractive healthy food offers
Regulation of packed lunches
Cooking, gardening and nutrition education
Welcoming dining environment
School caterers role as staff members
Availability of water
Children and staff eating in canteen
Tasters and theme days
Seeking pupils' views
Removal of foods as rewards
Widening access to healthy food
Consistent food messaging
School contextual factors: assessed through review of school policies, observations, school staff, governor, pupil and parent surveys, routine school data, case-studies)
School leadership/organisational culture
Influence of governors
Staff knowledge and skills
School engagement with parents
Physical school environment
School financial constraints
Socioeconomic / ethnic mix of pupils

Recruitment and data collection flowchart

RECRUITMENT

SCHOOL

Email: School invitation letters sent to school by email and hard copy

↓ >7 DAYS

Telephone call: Telephone call to follow up with schools on the email invitation

↓

Email: Send contract to interested schools prior to initial visit

↓

Recruitment visit: Attend school to complete recruitment process—sign school contract, identify study liaison, agree a sampling approach for classes with schools, identify difficult times in the school term and agree timeframe, discuss study promotion through social media channels

↓

PUPIL/STAFF

Parents: Send parent information sheet and opt-out form (hard copy, email or text) to parents of year 7, 9 and 10 pupils

Pupils: Teachers distribute pupil information sheet to year 7, 9 and 10 pupils (hard copy)

↓ >7 DAYS

CONSENT AND ASSENTTAKING

Opt out: Collect parental opt-out consent forms collected through study liaison staff member

DATA COLLECTION

Staff and Governors: Send staff information sheet, consent form and survey (hard copy and online)

↓

Request school data: Key information survey, costings survey and other school data and documents e.g. pupil ethnicity data, relevant curriculum documents, school menus

Pupil data collection (session 1) and food services observations visit(s): Attend school— observation of all school food service and dining areas across all eating occasions. Pupil data collection session 1: pupil assent, survey 1 and Intake24 collection 1 with all 3 year groups

Parent survey: Consent form and survey hard copy sent home with pupils at session 1 and online survey link emailed by school

↓ >7 DAYS

Pupil data collection session 2: Pupil survey 2 and Intake24 collection 2 (staff-led; no researcher present)

Parent survey reminder: School sends reminder via email or text system

Staff survey reminder: Research team sends reminder via email

Vouchers: Pupil vouchers distributed via school following session 2. Parent vouchers sent via post >2 weeks after second reminder.

For peer review only - http://bmjopen.bmj.com/site/about/guidelines.xhtml

SPIRIT

STANDARD PROTOCOL ITEMS: RECOMMENDATIONS FOR INTERVENTIONAL TRIALS

SPIRIT 2013 Checklist: Recommended items to address in a clinical trial protocol and related documents*

Section/item	Item No	Description	Addressed on page number
**Administrative information**			
Title	1	Descriptive title identifying the study design, population, interventions, and, if applicable, trial acronym	1
Trial registration	2a	Trial identifier and registry name. If not yet registered, name of intended registry	2 & 3
	2b	All items from the World Health Organization Trial Registration Data Set	1-15
Protocol version	3	Date and version identifier	2
Funding	4	Sources and types of financial, material, and other support	15
Roles and responsibilities	5a	Names, affiliations, and roles of protocol contributors	1 & 15
	5b	Name and contact information for the trial sponsor	14
	5c	Role of study sponsor and funders, if any, in study design; collection, management, analysis, and interpretation of data; writing of the report; and the decision to submit the report for publication, including whether they will have ultimate authority over any of these activities	15
	5d	Composition, roles, and responsibilities of the coordinating centre, steering committee, endpoint adjudication committee, data management team, and other individuals or groups overseeing the trial, if applicable (see Item 21a for data monitoring committee)	14

For peer review only

**Introduction**			
Background and rationale	6a	Description of research question and justification for undertaking the trial, including summary of relevant studies (published and unpublished) examining benefits and harms for each intervention	5-6
	6b	Explanation for choice of comparators	6
Objectives	7	Specific objectives or hypotheses	6
Trial design	8	Description of trial design including type of trial (eg, parallel group, crossover, factorial, single group), allocation ratio, and framework (eg, superiority, equivalence, noninferiority, exploratory)	6
**Methods: Participants, interventions, and outcomes**			
Study setting	9	Description of study settings (eg, community clinic, academic hospital) and list of countries where data will be collected. Reference to where list of study sites can be obtained	6
Eligibility criteria	10	Inclusion and exclusion criteria for participants. If applicable, eligibility criteria for study centres and individuals who will perform the interventions (eg, surgeons, psychotherapists)	7-8
Interventions	11a	Interventions for each group with sufficient detail to allow replication, including how and when they will be administered	N/A
	11b	Criteria for discontinuing or modifying allocated interventions for a given trial participant (eg, drug dose change in response to harms, participant request, or improving/worsening disease)	N/A
	11c	Strategies to improve adherence to intervention protocols, and any procedures for monitoring adherence (eg, drug tablet return, laboratory tests)	10
	11d	Relevant concomitant care and interventions that are permitted or prohibited during the trial	N/A
Outcomes	12	Primary, secondary, and other outcomes, including the specific measurement variable (eg, systolic blood pressure), analysis metric (eg, change from baseline, final value, time to event), method of aggregation (eg, median, proportion), and time point for each outcome. Explanation of the clinical relevance of chosen efficacy and harm outcomes is strongly recommended	10-11

For peer review only - http://bmjopen.bmj.com/site/about/guidelines.xhtml

Participant timeline	13	Time schedule of enrolment, interventions (including any run-ins and washouts), assessments, and visits for participants. A schematic diagram is highly recommended (see Figure)	8, 10, Supplementary file 1
Sample size	14	Estimated number of participants needed to achieve study objectives and how it was determined, including clinical and statistical assumptions supporting any sample size calculations	7
Recruitment	15	Strategies for achieving adequate participant enrolment to reach target sample size	7-8
**Methods: Assignment of interventions (for controlled trials) – N/A**			
Allocation:			
Sequence generation	16a	Method of generating the allocation sequence (eg, computer-generated random numbers), and list of any factors for stratification. To reduce predictability of a random sequence, details of any planned restriction (eg, blocking) should be provided in a separate document that is unavailable to those who enrol participants or assign interventions	N/A
Allocation concealment mechanism	16b	Mechanism of implementing the allocation sequence (eg, central telephone; sequentially numbered, opaque, sealed envelopes), describing any steps to conceal the sequence until interventions are assigned	N/A
Implementation	16c	Who will generate the allocation sequence, who will enrol participants, and who will assign participants to interventions	N/A
Blinding (masking)	17a	Who will be blinded after assignment to interventions (eg, trial participants, care providers, outcome assessors, data analysts), and how	N/A
	17b	If blinded, circumstances under which unblinding is permissible, and procedure for revealing a participant's allocated intervention during the trial	N/A
**Methods: Data collection, management, and analysis**			
Data collection methods	18a	Plans for assessment and collection of outcome, baseline, and other trial data, including any related processes to promote data quality (eg, duplicate measurements, training of assessors) and a description of study instruments (eg, questionnaires, laboratory tests) along with their reliability and validity, if known. Reference to where data collection forms can be found, if not in the protocol	8-11

For peer review only - http://bmjopen.bmj.com/site/about/guidelines.xhtml

	18b	Plans to promote participant retention and complete follow-up, including list of any outcome data to be collected for participants who discontinue or deviate from intervention protocols	10-11
Data management	19	Plans for data entry, coding, security, and storage, including any related processes to promote data quality (eg, double data entry; range checks for data values). Reference to where details of data management procedures can be found, if not in the protocol	11, 13, 14
Statistical methods	20a	Statistical methods for analysing primary and secondary outcomes. Reference to where other details of the statistical analysis plan can be found, if not in the protocol	12
	20b	Methods for any additional analyses (eg, subgroup and adjusted analyses)	12
	20c	Definition of analysis population relating to protocol non-adherence (eg, as randomised analysis), and any statistical methods to handle missing data (eg, multiple imputation)	12
**Methods: Monitoring**			
Data monitoring	21a	Composition of data monitoring committee (DMC); summary of its role and reporting structure; statement of whether it is independent from the sponsor and competing interests; and reference to where further details about its charter can be found, if not in the protocol. Alternatively, an explanation of why a DMC is not needed	N/A
	21b	Description of any interim analyses and stopping guidelines, including who will have access to these interim results and make the final decision to terminate the trial	N/A
Harms	22	Plans for collecting, assessing, reporting, and managing solicited and spontaneously reported adverse events and other unintended effects of trial interventions or trial conduct	N/A
Auditing	23	Frequency and procedures for auditing trial conduct, if any, and whether the process will be independent from investigators and the sponsor	N/A
**Ethics and dissemination**			
Research ethics approval	24	Plans for seeking research ethics committee/institutional review board (REC/IRB) approval	14

Protocol amendments	25	Plans for communicating important protocol modifications (eg, changes to eligibility criteria, outcomes, analyses) to relevant parties (eg, investigators, REC/IRBs, trial participants, trial registries, journals, regulators)	14
Consent or assent	26a	Who will obtain informed consent or assent from potential trial participants or authorised surrogates, and how (see Item 32)	7-8
	26b	Additional consent provisions for collection and use of participant data and biological specimens in ancillary studies, if applicable	N/A
Confidentiality	27	How personal information about potential and enrolled participants will be collected, shared, and maintained in order to protect confidentiality before, during, and after the trial	14
Declaration of interests	28	Financial and other competing interests for principal investigators for the overall trial and each study site	15-16
Access to data	29	Statement of who will have access to the final trial dataset, and disclosure of contractual agreements that limit such access for investigators	N/A
Ancillary and post-trial care	30	Provisions, if any, for ancillary and post-trial care, and for compensation to those who suffer harm from trial participation	N/A
Dissemination policy	31a	Plans for investigators and sponsor to communicate trial results to participants, healthcare professionals, the public, and other relevant groups (eg, via publication, reporting in results databases, or other data sharing arrangements), including any publication restrictions	14
	31b	Authorship eligibility guidelines and any intended use of professional writers	N/A
	31c	Plans, if any, for granting public access to the full protocol, participant-level dataset, and statistical code	14
**Appendices**			
Informed consent materials	32	Model consent form and other related documentation given to participants and authorised surrogates	
Biological specimens	33	Plans for collection, laboratory evaluation, and storage of biological specimens for genetic or molecular analysis in the current trial and for future use in ancillary studies, if applicable	N/A

For peer review only

*It is strongly recommended that this checklist be read in conjunction with the SPIRIT 2013 Explanation & Elaboration for important clarification on the items. Amendments to the protocol should be tracked and dated. The SPIRIT checklist is copyrighted by the SPIRIT Group under the Creative Commons "Attribution-NonCommercial-NoDerivs 3.0 Unported" license.

For peer review only

BMJ Open

The Food provision, cUlture and Environment in secondary schooLs (FUEL) study: protocol of a mixed methods evaluation of national School Food Standards implementation in secondary schools and their impact on pupils' dietary intake and dental health

Journal:	*BMJ Open*
Manuscript ID	bmjopen-2020-042931.R1
Article Type:	Protocol
Date Submitted by the Author:	17-Aug-2020
Complete List of Authors:	Murphy, Marie; University of Birmingham, Institute of Applied Health Research<br>Pallan, Miranda; University of Birmingham, Institute of Applied Health Research<br>Lancashire, Emma; University of Birmingham, Institute of Applied Health Research<br>Duff, Rhona; University of Birmingham, Institute of Applied Health Research<br>Adamson, Ashley; Newcastle University, Human Nutrition Research CentreInstitute of Health & Society<br>Bartington, Suzanne; University of Birmingham, Institute of Applied Health Research<br>Frew, Emma; University of Birmingham, Institute of Applied Health Research<br>Griffin, Tania ; University of Bath,<br>Hurley, Kiya; University of Birmingham, Cancer Research UK Clinical Trials Unit<br>Parry, Jayne; University of Birmingham, Institute of Applied Health Research<br>Passmore, S; Services for Education Ltd,<br>Ravaghi, Vahid; University of Birmingham, School of Dentistry<br>Sitch, Alice; University of Birmingham, Institute of Applied Health Research; University Hospitals Birmingham NHS Foundation Trust, NIHR Birmingham Biomedical Research Centre<br>Spence, Suzanne; Newcastle University, Institute of Health & Society, Human Nutrition Research Centre<br>Rowland, Maisie; Newcastle University, Human Nutrition Research Centre and Population Health Sciences Institute<br>Wheeldon, Scott; Wheelers Lane Technology College<br>Adab, Peymane; University of Birmingham, Institute of Applied Health Research
**Primary Subject Heading**:	Public health
Secondary Subject Heading:	Nutrition and metabolism, Epidemiology

Keywords:	EPIDEMIOLOGY, PUBLIC HEALTH, NUTRITION & DIETETICS

SCHOLARONE™
Manuscripts

I, the Submitting Author has the right to grant and does grant on behalf of all authors of the Work (as defined in the below author licence), an exclusive licence and/or a non-exclusive licence for contributions from authors who are: i) UK Crown employees; ii) where BMJ has agreed a CC-BY licence shall apply, and/or iii) in accordance with the terms applicable for US Federal Government officers or employees acting as part of their official duties; on a worldwide, perpetual, irrevocable, royalty-free basis to BMJ Publishing Group Ltd ("BMJ") its licensees and where the relevant Journal is co-owned by BMJ to the co-owners of the Journal, to publish the Work in this journal and any other BMJ products and to exploit all rights, as set out in our licence.

The Submitting Author accepts and understands that any supply made under these terms is made by BMJ to the Submitting Author unless you are acting as an employee on behalf of your employer or a postgraduate student of an affiliated institution which is paying any applicable article publishing charge ("APC") for Open Access articles. Where the Submitting Author wishes to make the Work available on an Open Access basis (and intends to pay the relevant APC), the terms of reuse of such Open Access shall be governed by a Creative Commons licence – details of these licences and which Creative Commons licence will apply to this Work are set out in our licence referred to above.

Other than as permitted in any relevant BMJ Author's Self Archiving Policies, I confirm this Work has not been accepted for publication elsewhere, is not being considered for publication elsewhere and does not duplicate material already published. I confirm all authors consent to publication of this Work and authorise the granting of this licence.

For peer review only

TITLE

The Food provision, cUlture and Environment in secondary schooLs (FUEL) study: protocol of a mixed methods evaluation of national School Food Standards implementation in secondary schools and their impact on pupils' dietary intake and dental health

AUTHORS

Marie Murphy[1] and Miranda Pallan[1] (joint first), Emma Lancashire[1], Rhona Duff[1], Ashley Adamson[2], Suzanne Bartington[1], Emma Frew[1], Tania Griffin[3], Kiya Hurley[4], Jayne Parry[1], Sandra Passmore[5], Vahid Ravaghi[6], Alice Sitch[1,7], Suzanne Spence[2], Maisie K Rowland[2], Scott Wheeldon[8], Peymane Adab[1]

1. Institute of Applied Health Research, University of Birmingham, Birmingham, UK
2. Human Nutrition Research Centre, Population Health Sciences Institute, Newcastle University, Newcastle, UK
3. Department for Health, University of Bath, Bath, UK
4. Cancer Clinical Trials Unit, University of Birmingham, Birmingham, UK
5. Services for Education, Birmingham, UK
6. School of Dentistry, University of Birmingham, Birmingham, UK
7. NIHR Birmingham Biomedical Research Centre, University Hospitals Birmingham NHS Foundation Trust and University of Birmingham, UK
8. Wheelers Lane Technology College, Birmingham, UK

Corresponding author

Miranda Pallan
Room 107, Murray Learning Centre
University of Birmingham
Edgbaston
Birmingham
UK
B15 2TT
Email: m.j.pallan@bham.ac.uk

Word count = 3,947 (excluding title page, abstract, contributions, acknowledgements, references, figures and tables)

For peer review only - http://bmjopen.bmj.com/site/about/guidelines.xhtml

KEY WORDS:

Food policy

Schools

Adolescents

Dietary intake

Dental health

ABBREVIATIONS

BAME = Black, Asian and Minority Ethnic

CHU-9D = Child Health Utility 9 Dimension questionnaire

DfE = Department for Education

EAL = English As a foreign Language

FG = Focus Group

FSM = Free School Meals

ICC = Intraclass-cluster Correlation Coefficient

IDACI = Income Deprivation Affecting Children Index

IMD = Index of Multiple Deprivation

ISRCTN = International Standard Randomised Controlled Trials Number

MAT = Multi-Academy Trust

NDNS = National Diet and Nutrition Survey

NPT = Normalization Process Theory

QALY = Quality Adjusted Life Year

SD = Standard Deviation

SEN = Special educational needs

SFS = School Food Standards

SFP = School Food Plan

SSC = Study Steering Committee

UK = United Kingdom

REGISTRATION

ISRCTN: 68757496 (registered 17th October 2019)

Protocol version 5.0 (1st May 2020)

For peer review only - http://bmjopen.bmj.com/site/about/guidelines.xhtml

ABSTRACT

Introduction: Excess free sugar intake is associated with obesity and poor dental health. Adolescents consume substantially more free sugar than is recommended. National (UK) School Food Standards (SFS) are in place, but are not mandatory in all schools, and their impact on the diets of secondary school pupils is unknown. We aim to evaluate how SFS and wider healthy eating recommendations (from the national School Food Plan (SFP)) are implemented in secondary schools, and how they influence pupils' diets and dental health.

Methods and analysis: Secondary-level academies/free schools in the West Midlands, UK were divided into two groups: SFS mandated; SFS non-mandated. Using propensity scores to guide sampling, we aim to recruit 22 schools in each group. We will compare data on school food provision and sales, school food culture and environment, and the food curriculum from each group, collected through: school staff, governor, pupil, parent surveys; school documents; and observation. We will explore implementation level for the SFS requirements and SFP recommendations, and develop a school food typology. We aim to recruit 1980 pupils aged 11-15 years across the 44 schools and collect dietary intake (24 hour recall) and dental health data through self-completion surveys. We will compare free sugar/other dietary intake and dental health across the two SFS groups, and across the identified school types. School type will be further characterised in 4-8 case study schools through school staff interviews and pupil focus groups. Evaluation of economic impact will be through a cost-consequence analysis and an exploratory cost-utility analysis.

Ethics and dissemination: Ethical approval was obtained from the University of Birmingham Ethical Review Committee (ERN_18-1738). Findings will be disseminated to key national and local agencies, schools and the public through reports, presentations, the media and open access publications.

Registration details: ISRCTN 68757496 (registered 17/10/2019)

For peer review only

For peer review only - http://bmjopen.bmj.com/site/about/guidelines.xhtml

ARTICLE SUMMARY

Strengths and limitations of this study

1. This research fills a gap in the literature by evaluating the impact of national school food policy upon the dietary intake of secondary school pupils in the UK.
2. A validated online dietary assessment tool, adapted for use in an ethnically diverse population, will be used with 1980 secondary school pupils across 44 schools.
3. The research will assess variation in the implementation of the School Food Standards, School Food Plan recommendations and other contextual factors across schools.
4. The study design includes qualitative research to provide an in-depth understanding of school food provision, environments and the culture/ethos relating to this.
5. The study aims to compare schools that are mandated, with schools that are not mandated to adhere to the School Food Standards. These two groups may differ in other ways, so to improve comparability of the two groups, a sampling approach based on propensity scores has been conducted.

For peer review only

For peer review only - http://bmjopen.bmj.com/site/about/guidelines.xhtml

MAIN TEXT

INTRODUCTION

Excess sugar consumption is a major contributor to increased energy intake/obesity, adverse cardio-metabolic health,[1] and poor dental health.[2] UK adolescents consume three times their recommended amount of total energy intake from free sugars;[3] almost half of 15 year olds have dental caries;[2] and nearly a third have excess weight.[4] Adolescence is an important time for dietary intervention as it is a key period for establishing dietary patterns,[5] with greater autonomy over dietary decisions. A large proportion of adolescent dietary intake occurs whilst at school, making these opportune settings for intervention.

In the UK, a longstanding strategy for improving children's diet has been nutritional standards for school food. School meal standards were first introduced in 1941, but fell out of favour later in the 20th century. In 2006, following a national school meals review,[6] national School Food Standards (SFS) were relaunched in England and became a legal requirement for most state schools. In 2015, a Department for Education (DfE) SFS review resulted in substantial changes. For ease of implementation, nutrient-based standards were removed whilst retaining food-based standards underpinned by a nutrient framework.[7]

In addition to the national SFS, the School Food Plan (SFP) was launched in 2013, providing a wider set of non-statutory recommendations for schools that promote a 'whole school' approach to healthy eating.[8] One central aim was to increase school meal uptake, as higher demand enables better quality meals to be served at lower cost. Another aim was to provide practical support, advice and information for headteachers to help improve quality and uptake of their school's food. In addition to school food provision, the plan addresses how healthy eating can be incorporated within all aspects of school life and the wider community.[9]

Evaluation of the impact of the 2006 SFS on food provision and consumption has been conducted in primary (age 4-11 years) and middle (age 9-12 years) schools in the UK. Pre- and post- SFS implementation comparisons showed improvements in overall dietary intake in pupils aged 4-7 years, but not in those aged 11-12 years.[10-12] SFS implementation was good in primary schools but less so in middle schools.[10] Evidence of SFS impact on secondary school pupils' (aged 11-16 years) dietary intake is more limited. A study in 80 secondary schools compared school food provision in 2011 with that in 2004. They reported improvements in nutritional content of school-provided food (in particular reduced

For peer review only - http://bmjopen.bmj.com/site/about/guidelines.xhtml

confectionary availability) and pupils' lunchtime food consumption, but did not examine total dietary intake.[13]

To date, the impact of the updated SFS or the SFP has not been evaluated. The way in which SFS legislation was introduced in England means that certain school types (including academies and free schools; 70% of secondary schools in England)[14] which were set up between January 2010 and May 2014 are exempt from this legislation (although they can choose to voluntarily sign up to the standards), whereas schools of these types established before or after these dates are legally required to meet the SFS. This provides an opportunity to examine how a legal requirement to meet the SFS influences schools and their pupils. Given the lack of SFS /SFP evaluation in secondary schools, we aim to investigate their influence in these settings by comparing school food provision, sales, wider school factors related to food, pupil dietary intake and dental health across those mandated and not mandated to adhere to the current SFS. We will also explore variation in implementation of the SFS and SFP recommendations, and their economic impact.

METHODS AND ANALYSIS

This is an observational, mixed method design study, consisting of two phases. Phase 1 involves collecting a variety of data on SFS/SFP implementation, and school and pupil outcomes in the SFS mandated and non-mandated schools. We will compare outcomes across the two groups and develop a typology of schools based on SFS/SFP implementation and wider school contextual factors. In phase 2, we will identify a small number of 'case study' schools and conduct a qualitative inquiry to further develop the school typology. Furthermore, we will undertake an economic evaluation to assess how the costs and outcomes vary by school typology.

Study setting

The sampling frame comprises secondary phase academies/free schools providing education to children aged 11-16 years and located within 14 Local Authority areas in the West Midlands region, UK. This region has a population of five million (21% of ethnicity other than White British),[15] urban, suburban and rural areas, and areas of high socioeconomic deprivation.[16] Other school types and academies that provide specialist alternative education have been excluded.

Phase 1

For peer review only - http://bmjopen.bmj.com/site/about/guidelines.xhtml

Sampling

To increase comparability of the two school groups, stratified sampling, based on propensity scores, was used.[17] We obtained routine data from the Department for Education on several characteristics for all schools in the sampling frame: Local Authority area; establishment type; urban/rural; total pupil roll size; Income Deprivation Affecting Children Index (IDACI); inclusion of a sixth form; selective/non-selective admissions policy; religious affiliation/secular; and proportion of: male/female pupils; pupils from Black, Asian and minority ethnic groups (BAME); students with English as a foreign language (EAL); students eligible for free school meals (FSM); and pupils with Special Educational Needs (SEN). We developed propensity scores using linear regression with the SFS status of the school (mandated/non-mandated) as the outcome and school characteristics as explanatory variables. Propensity score quartiles were then used to create four groups with subsequent division by SFS status (based on the date they received academy/free school status), resulting in eight distinct sampling groups. Following random ordering of each group, schools will be invited sequentially to participate, aiming to recruit five or six schools from each group.

Within participating schools, pupils in one class from each of years 7, 9 and 10 (aged 11-12, 13-14 and 14-15 years, respectively) will be invited to take part. Preference will be given to classes not streamed by academic ability or subject to enable participation of classes which are representative of the year group characteristics. There are no pupil exclusion criteria.

Sample size calculation

We used data on free sugar intake pre- and post-SFS implementation from the study undertaken in middle schools by Adamson et al.[10] to inform our sample size calculation. Assuming an intra-class correlation coefficient (ICC) of 0.1 [18] and balanced cluster sizes, we estimated that to detect a difference in mean free sugar intake at lunch of 4g (20g vs.16g) between the two school groups, assuming a SD of 11 with 90% power and at 5% significance, we require 990 evaluable participants and 22 clusters (conservatively using schools[19]) in each group (total schools=44; total participants n=1980; cluster size=45).

Recruitment

Headteachers will be invited by post and email with telephone follow-up. In participating schools a liaison staff member will be identified and a contract, outlining expected commitments from both parties, will be signed. Once data collection is complete, participating schools will receive a school-specific summary report and £300.

For peer review only - http://bmjopen.bmj.com/site/about/guidelines.xhtml

At least seven days prior to pupil data collection, pupils from the three selected classes will receive a participant information pack (comprising pupil and parent information sheets and a parental opt-out consent form). Schools will also be asked to e-mail documents directly to parents. Pupils whose parents do not return a completed opt-out consent form will be invited to take part in the data collection sessions during the school day and asked for their electronic assent for study participation. Parents of pupils in selected classes will be invited to complete a self-administered parent survey (online or paper copy). Both pupils and parents who participate receive a £5 voucher.

Participating schools will be asked to identify key staff/governors with roles relating to food provision, eating environment, food curriculum, or SFS/SFP implementation. Identified staff will be sent a staff participant information sheet (email/paper copy) and study invitation for a self-administered survey (online or paper format). Recruitment and data collection processes are outlined in Supplementary File 1.

School and pupil recruitment commenced in October and November 2019 respectively, and was due to be completed in the 2019/20 academic year. However, due to restrictions in place in England during the COVID-19 pandemic, recruitment and data collection was suspended in March 2020 and will recommence when schools re-open for all pupils to attend. All further data collection activities will be undertaken in accordance with relevant Department of Health and Social Care and Department for Education health protection guidance.

Data collection

Data capture on SFS and SFP implementation, and the wider school context

A logic model setting out the processes by which the SFS/SFP are assumed to generate pupil health gains has been developed (Figure 1). Briefly, we hypothesise health gain materialises directly via a change in school food consumption, and indirectly by curricular and other activities designed to change pupils' dietary knowledge, attitudes and beliefs which impact on food consumption in and out of school. The extent to which the SFS/SFP achieve these health gains depends on their implementation within a school, which is influenced by key contextual factors (e.g. school management, parental engagement etc.).

Our data collection strategy, guided by the principles for process evaluation outlined in the UK Medical Research Council guidance,[20] will provide data to populate and refine the logic model. This will enable us to assess the extent to which the SFS requirements and SFP

For peer review only - http://bmjopen.bmj.com/site/about/guidelines.xhtml

recommendations are embedded in each school, and the relative importance of contextual factors in influencing this. Our data collection methods include: school menu analysis; observation of school food provision and the related environment; school document review; collection of aggregated routine data on pupil characteristics; and self-administered surveys for staff, governors, pupils and parents.

Assessment of SFS compliance and SFP recommendations implementation

SFS compliance assessment is based on the national SFS checklists for school lunch and school food other than lunch [21] that consist of daily, weekly and three-weekly criteria for food and drink offered. Using an observation tool developed to measure compliance with the SFS daily criteria, trained researchers will record food and drink provision at all eating occasions across one school day, and at all school food outlets and dining areas. Compliance with the SFS weekly and three-weekly criteria will be assessed from schools' weekly menus.

Implementation of the SFP recommendations is based on three guidance documents produced by the DfE: 1) a headteacher checklist; 2) a guide to creating a culture and ethos of healthy eating; and 3) guidance for governors.[22-24] Implementation will be assessed through questions in the surveys to pupils, parents, staff and governors; researcher observation of food outlets and dining areas; and school document review, e.g. school food policy, catering contract, curriculum documents and minutes of the Board of Governors and School Council meetings.

The school staff and governor surveys include a series of questions based on Normalization Process Theory (NPT),[25] to explore the implementation and sustained embedding of the SFS/SFP. The items in the NoMAD instrument (developed to assess the four NPT constructs),[26] were adapted for relevance to the SFS/SFP, and the school setting.

Assessment of school lunch uptake and food sales

To assess school lunch uptake, schools will be asked to provide their routinely collected school meal uptake data. To assess school food sales, we will request weekly aggregated food sales data for two pre-specified months in the previous year. Most schools use online payment management systems, which provide sales data by types of food/drinks sold. We will extract data on the number of items sold for both SFS restricted food/drinks (including sugar sweetened beverages, confectionary, fried food, snacks etc.) and pre-defined healthy foods and compare sales of these food categories across schools, taking into account school size and school lunch uptake.

For peer review only - http://bmjopen.bmj.com/site/about/guidelines.xhtml

Assessment of school contextual factors

We will collect data on a variety of contextual factors through observation, school document review, and the staff, governor, pupil and parent surveys, with further exploration in the second phase case studies. These factors include: the physical school environment; school leadership and organisational culture; the influence of governors; staff knowledge and skills; school engagement with parents; and school financial considerations. In addition, socioeconomic and demographic characteristics of the pupil population will be assessed through routinely available school data. Information on SFS voluntary sign-up will also be requested from non-mandated schools.

Data capture on SFS/SFP resource use

Food provision costs will be estimated from a detailed list of resources obtained through a school staff survey, one-day researcher observation, catering contracts, and school menus and pricing. Wider costs associated with SFP implementation will be obtained from school staff, parent and pupil surveys and observation. Each resource use item will be costed using financial data supplied by the school, or obtained from published literature.

Pupil outcome data collection

We aim to compare dietary intake and dental health in pupils in the two defined SFS school groups. The primary outcomes are intake of free sugars (grams): i) during school day lunch (determined by asking pupils when they ate their lunch); ii) whilst at school; and iii) during the full 24 hour period of the same school day. Secondary dietary outcomes include: percentage of dietary energy intake from free sugars; total energy intake (kcal); total fat intake (grams); fibre intake (grams); number of sugar-sweetened beverages consumed; number of sugar and chocolate confectionary items consumed; number of foods high in fat, sugar and salt consumed; and number of fruit and vegetable portions consumed. These outcomes will also be compared across the two school groups for the three defined time-periods (school day lunch; whilst in school; over 24 hours). Additional secondary dietary outcomes are: free sugar intake providing >5% energy intake; number of eating/drinking occasions (excluding plain water); and consumption of five or more portions of fruit and vegetables per day. Secondary outcomes relating to dental health are: the presence of dental caries; the number of dental caries symptoms; and treatment received for dental caries.

Self-reported pupil data will be collected in school, during two timetabled sessions approximately 1-4 weeks apart. Pupils will complete an online survey at each session followed by a 24 hour recall dietary assessment. The first and second sessions will be facilitated by

For peer review only - http://bmjopen.bmj.com/site/about/guidelines.xhtml

trained researchers and classroom teachers, respectively. An alternative activity will be provided for non-participating pupils.

Dietary intake assessment:

Dietary intake will be measured using Intake24, an online self-completion 24 hour dietary recall tool based on the multiple pass method, which has been shown to be the most accurate for assessing adolescent dietary intake.[27] Compared with interviewer-led recall, Intake24 under-estimated energy intake by just 1% in this age group, and differences in mean macro-/micro-nutrient intakes between the two methods were within 4%.[28]

Nutrient analysis of Intake24 data uses the National Diet and Nutrition Survey (NDNS) food database containing over 2300 foods linked to the UK Nutrient Databank codes.[29] Intake24 piloting, community nutritionist consultation and literature review identified food/drinks commonly consumed by minority ethnic groups but not included in this database (n=63), which were added to Intake24. Nutritional data for these items were obtained through matching to existing items in Intake24 or from other existing food composition sources.[30, 31] Photographs are used for portion size estimation, a method which has shown good agreement with four day weighed intakes in adolescents.[32, 33]

Assessment of dental caries experience:

We will use validated self-report measures from the national Child Dental Health Survey [2] to assess dental caries symptoms in the last 3 months and treatment received in the last 24 months. Self-reported tooth brushing frequency data will also be collected.

Other pupil data collection:

Pupils will be asked to provide their age, sex, ethnicity, postcode (for mapping to Index of Multiple Deprivation (IMD) scores and exposure to fluoridated water), usual lunch type (school-provided vs. home-packed), usual mode of travel to/from school, and sleep/wake times for the previous night. Pupils will also complete the Child-Health Utilities 9-Dimension (CHU-9D), a utility-based health-related quality of life questionnaire,[34] for use within the economic evaluation.

Data collection tool piloting

The school staff, governor, pupil and parent surveys, the adapted Intake24 and the observation tool were piloted with the relevant target groups. The tools were revised in line with the feedback obtained.

For peer review only - http://bmjopen.bmj.com/site/about/guidelines.xhtml

Data analysis

Preliminary development of a school typology

Using the logic model (Figure 1) as a guide, we will use data captured on SFS/SFP implementation and the school context to develop an initial typology of schools, which will reflect the degree of implementation of the SFS/SFP and other relevant local initiatives relating to food and healthy eating.

Analysis of pupil outcomes

Pupils will have a maximum of two 24 hour recalls for dietary intake measures. To summarise dietary outcomes, we will report the average value across the two days for each pupil. If only a single day is available, we will use data from that day only. Differences between the SFS mandated and SFS non-mandated school groups for both primary and secondary pupil outcomes will be assessed using multilevel linear models that account for clustering at the school and class level (and at the Multi-Academy Trust level if applicable). Models will include adjustment for propensity scores and pupil-level covariates (school year group, sex, ethnicity, IMD score and school-provided vs home-packed lunch). Models for dietary outcomes will use data recorded each day, allowing for the repeated outcome measures. Models for dental outcomes will be additionally adjusted for oral healthcare factors and water fluoridation exposure. We will also explore whether the associations between school SFS status and pupil outcomes differ across age, socioeconomic position (indicated by IMD score), or usual lunch type (school-provided vs. home-packed) by adding the relevant interaction terms to the developed models. If more than 5% of data are missing from demographic variables, multiple imputation methods will be used and sensitivity analyses conducted.

If possible, within the group of schools not-mandated to adhere to the SFS, we will undertake an exploratory analysis to examine differences in pupil outcomes between schools who have and have not voluntarily signed up to the SFS. In addition, we will develop further linear multilevel models to explore potential associations between the identified school types and pupil outcomes, again adjusting for clustering, potential pupil-level confounders and propensity scores, as described above.

Phase 2: school case studies

A number of schools (n=4-8) will be asked to participate in a qualitative study. Schools will be selected to comprise a range of school types and include at least two schools from the SFS non-mandated group not reporting voluntary sign-up. These schools will receive a further £150.

For peer review only - http://bmjopen.bmj.com/site/about/guidelines.xhtml

We will undertake interviews with key school stakeholders (identified by the school Senior Leadership Team, aiming for 4-6 per school) to explore in more depth the way in which the SFS/SFP and local school policy or initiatives are introduced, embedded and sustained in the schools, and their perceived influence on the dietary intake of pupils. Data collection will be shaped with reference to the four constructs, and their constituent components, within May's NPT[25] and by Maguire's exploration of policy enactment in English schools.[35] Interviewees will be encouraged to tell their 'story' relating to their experiences of the SFS/SFP; how the provision of food, eating environments and the food/cooking curriculum have been shaped within their schools; and the influence of the wider school context.

Through focus groups (FGs) with school pupils (aiming for one FG in each included year group per school, comprising 6-8 participants per FG) we will explore their views of the school food environment, contextual factors influencing this, and how they interact with this and the wider external environment surrounding the school, in terms of their eating behaviour. We will also explore views and experiences of any negative impact of SFS/SFP and regulation of foods provided. Participating pupils will receive a £5 voucher.

Interviews/FGs will be audio-recorded with participant consent, transcribed ad verbatim and anonymised. We will use the framework analytical approach,[36] and within this undertake thematic analysis, guided by NPT and Maguire's policy enactment exploration.[25, 35]

Economic evaluation

To evaluate economic impact of the SFS and the wider SFP a cost-consequence analysis will be undertaken. We will summarise costs and outcomes in the form of a balance sheet. The analysis will highlight the costs to schools and families, and offer a transparent range of outcome measures for consideration. In addition, we will conduct an exploratory cost-utility analysis. Quality-Adjusted Life Years (QALYs) will be constructed from pupil responses to CHU-9D to allow inferences to be made about QALY-differences between the two school groups offset against cost.

Patient and public involvement

During research plan development, the deputy headteacher member of the investigator team advised on engaging schools and pupils and access to food sales data, and provided background information on school management and governance systems. In addition we consulted with a group of parents of secondary school children, a group of secondary school pupils, a teacher and

For peer review only - http://bmjopen.bmj.com/site/about/guidelines.xhtml

For peer review only

a manager of a school catering company, who advised on school, pupil and parent recruitment, data collection methods and survey content. To provide ongoing advice from a public perspective, we have convened a group of parents, teachers and secondary school governors (n=8), and two groups of secondary school pupils to consult with at key points throughout the study. To date these groups have reviewed and piloted surveys and Intake24, and advised on: developing a safeguarding policy; school, staff, pupil and parent recruitment and school retention; data collection practicalities; and the alternative activity for non-participating pupils.

ETHICS AND DISSEMINATION

Full ethical approval for the study was obtained from the University of Birmingham Science, Technology, Engineering and Mathematics Ethical Review Committee on 20th August 2019 (ERN_18-1738).

Data management and study oversight

The University of Birmingham is the Study Sponsor and data controller and assumes overall responsibility for the study. Data management and storage is compliant with the UK Data Protection Act 2018 and follows relevant University of Birmingham policy and procedures. Anonymised data will be stored securely for a minimum of 10 years after the publication of the main study results.

An independently chaired Study Steering Committee (SSC) has been convened to provide study oversight. Membership comprises three independent academics with relevant expertise, a representative from Public Health England (PHE), a public representative and the Chief Investigator. The Committee has agreed the current protocol and will review subsequent amendments.

Dissemination

Study findings will be disseminated to key national agencies (e.g. Department of Health and Social Care, DfE, PHE, School Food Alliance, National Association for Headteachers, LACA (a national umbrella organisation for school food caterers)), local level organisations (e.g. Regional School Commissioners and Local Authorities), and schools through reports, conference/meeting presentations, the educational and general press and open access publications. The public representatives on the Investigator team, SSC and other public engagement groups will advise further on dissemination plans for the study findings. A full report of the study will be published

For peer review only - http://bmjopen.bmj.com/site/about/guidelines.xhtml

in the NIHR Journals Library. After publication of the main study findings, anonymised data will be available on request from the study Chief Investigators.

AUTHOR CONTRIBUTIONS

MP/PA are the Chief Investigators with overall responsibility for the study. They conceived the study with input from SB and EL. MP and PA led on protocol development with contribution from all named authors. MP, PA, EL, MM, and RD developed the data collection tools. AA and MKR designed the dietary data collection tool Intake24, and RD, MKR, MM and MP contributed to the adaptation and piloting of Intake24 for the purposes of this study. AS designed and conducted the sampling procedure, and developed the analysis plan. EF designed the economic evaluation. VR advised on dental outcome measures. JP advised on the case study methodology. SP and SW advised on recruiting and working within schools. SS advised on the procurement and analysis of school food sales data. TG and KH advised on a range of methodological and practical aspects of the study. MM, MP, EL and RD drafted the manuscript and all authors critically reviewed the manuscript and agreed the final draft.

FUNDING STATEMENT

This study is funded by the National Institute for Health Research (NIHR) Public Health Research programme (17/92/39). Alice Sitch is part-funded by the NIHR Birmingham Biomedical Research Centre at the University Hospitals Birmingham NHS Foundation Trust. The views expressed are those of the authors and not necessarily those of the NIHR, NHS or the Department of Health and Social Care.

ACKNOWLEDGMENTS

Thank you to our Study Steering Group members Sheila Birdi, Helen Carter, Ann Hughes, Christopher Owen, Alexander Turner and Robert West (Chair) for their guidance. We are grateful for the contributions that our public advisors have made, including pupils and teachers at two schools in the West Midlands. We are also grateful to all those individuals who piloted study materials or provided advice on food/drink items commonly consumed by minority ethnic groups in the West Midlands.

For peer review only - http://bmjopen.bmj.com/site/about/guidelines.xhtml

For peer review only

COMPETING INTERESTS STATEMENT

Miranda Pallan, Emma Lancashire, Ashley Adamson, Suzanne Bartington, Emma Frew, Tania Griffin, Kiya Hurley, Jayne Parry, Sandra Passmore, Vahid Ravaghi, Alice Sitch and Peymane Adab hold grants from the UK National Institute for Health Research (NIHR). Peymane Adab is Chair of the NIHR Public Health Research Funding Committee. Ashley Adamson is Director of the NIHR School for Public Health Research, a NIHR Senior Investigator and a member of both the School Food Plan Alliance and UK Prevention Research Partnership network Generating Excellent Nutrition in UK Schools (GENIUS). Jayne Parry is Chair of a NIHR Fellowships selection committee. Alice Sitch is a grant holder for the Birmingham NIHR Biomedical Research Centre. Suzanne Bartington is an elected member of Oxfordshire County Council. There are no other competing interests to declare.

REFERENCES

1. Johnson RK, Appel LJ, Brands M, et al. Dietary sugars intake and cardiovascular health: a scientific statement from the American Heart Association. *Circulation* 2009;120:1011-1020.

2. Holmes R, Porter J, Vernazza C, et al. Children's Dental Health Survey 2013. Country Specific Report: England, 2015.

3. Bates B, Cox L, Nicholson S, et al. Results of the National Diet and Nutrition Survey (NDNS) rolling programme for 2012 to 2013 and 2013 to 2014. London, 2016.

4. Conolly A: Health Survey for England 2015: Children's body mass index, overweight and obesity. Leeds, 2016.

5. Wang Y, Bentley ME, Zhai F, et al. Tracking of dietary intake patterns of Chinese from childhood to adolescence over a six-year follow-up period. *J Nutr* 2002;132:430-38.

6. Hope P. Statutory Instrument 2006 No. 2381 The Education (Nutritional Standards for School Lunches) (England) Regulations. London, 2006.

7. Mucavele P, Nicholas J, Sharp L. Development and pilot testing of revised food-based standards for school lunches in England. Sheffield: Children's Food Trust,; 2013. Available from: https://www.schoolfoodplan.com/wp-content/uploads/2014/02/School-Food-Plan-Pilot-study-EVALUATION-REPORT-Final-V3.pdf accessed June 2019.

8. The School Food Plan. The Independent School Food Plan 2019. Available from: http://www.schoolfoodplan.com/ accessed June 2019.

9. Dimbleby H, Vincent J. The School Food Plan 201. Available from: http://www.schoolfoodplan.com/wp-content/uploads/2013/07/School_Food_Plan_2013.pdf accessed May 2020.

10. Adamson AJ, White M, Stead M. The process and impact of change in the school food policy on food and nutrient intake of children aged 4-7 and 11-12 years both in and out of school: a mixed methods approach: Public Health Research Consortium 2012.

For peer review only - http://bmjopen.bmj.com/site/about/guidelines.xhtml

11. Spence S, Delve J, Stamp E, et al. The impact of food and nutrient-based standards on primary school children's lunch and total dietary intake: a natural experimental evaluation of government policy in England. *PloS ONE* 2013;8:e78298.

12. Spence S, Matthews JN, White M, et al. A repeat cross-sectional study examining the equitable impact of nutritional standards for school lunches in England in 2008 on the diets of 4-7y olds across the socio-economic spectrum. *Int J Behav Nutr Phys Act* 2014;11:128.

13. Nicholas J, Wood L, Harper C, et al. The impact of the food-based and nutrient-based standards on lunchtime food and drink provision and consumption in secondary schools in England. *Public Health Nutr* 2013;16:1052-65.

14. Morse A. Converting maintained schools to academies. London: National Audit Office,, 2018.

15. Gov.uk. Regional ethnic diversity 2018. Available from: https://www.ethnicity-facts-figures.service.gov.uk/uk-population-by-ethnicity/national-and-regional-populations/regional-ethnic-diversity/latest#areas-of-england-and-wales-by-ethnicity accessed April 2020.

16. Medland A. Portrait of the West Midlands. In: Office for National Statistics, ed., 2011.

17. Rubin DB. Estimating causal effects from large data sets using propensity scores. *Ann Intern Med* 1997;127:757-63.

18. Juras R. Estimates of Intraclass Correlation Coefficients and Other Design Parameters for Studies of School-Based Nutritional Interventions. *Eval Rev* 2016;40:314-33.

19. Hemming K, Lilford R, Girling AJ: Stepped-wedge cluster randomised controlled trials: a generic framework including parallel and multiple-level designs. *Stat Med* 2015;34:181-196.

20. Moore G, Audrey S, Barker M, et al. Process evaluation of complex interventions: a summary of Medical Research Council guidance. . In: Richards D, Hallberg IR, eds. Complex interventions in health: an overview of research methods. Abingdon: Routledge, 2015.

21. The School Food Plan. School food standards: a practical guide for schools their cooks and caterers 2016. Available from: http://www.schoolfoodplan.com/wp-content/uploads/2014/06/School-Food-Standards-Guidance-FINAL-V1a-140616.pdf accessed June 2019.

22. The School Food Plan. A checklist for head teachers 2013. Available from: https://www.schoolfoodmatters.org/sites/default/files/SFP-Headteacher-Checklist_0.pdf accessed June 2019.

23. The School Food Plan. Creating a Culture and Ethos of Healthy Eating 2013. Available from: http://www.schoolfoodplan.com/wp-content/uploads/2015/09/Creating-a-culture-ethos-of-healthy-eating-Practical-Guidance-Final.pdf accessed June 2019.

24. The School Food Plan. School Food: Guidance for Governors 2013. Available from: http://www.schoolfoodplan.com/wp-content/uploads/2016/03/School-Governors_Guidance.pdf accessed June 2019.

25. May C, Finch T. Implementing, Embedding, and Integrating Practices: An Outline of Normalization Process Theory. *Sociology* 2009;43:535-54.

For peer review only

26. Finch TL, Girling M, May CR, et al. NoMad: Implementation measure based on Normalization Process Theory. 2015. Available from: http://www.normalizationprocess.org accessed November 2019.

27. Burrows TL, Martin RJ, Collins CE. A systematic review of the validity of dietary assessment methods in children when compared with the method of doubly labeled water. *J Am Diet Assoc* 2010;110:1501-10.

28. Bradley J, Simpson E, Poliakov I, et al. Comparison of INTAKE24 (an Online 24-h Dietary Recall Tool) with Interviewer-Led 24-h Recall in 11-24 Year-Old. *Nutrients* 2016;8:358.

29. Roe M, Pinchen H, Church S, et al. McCance and Widdowson's the composition of foods seventh summary edition and updated composition of foods integrated dataset. *Nutr Bull* 2015;40:36-39.

30. Stadlmayr B, Charrondiere UR, Enujiugha VN, et al. West African Food Composition Table. Available from: http://www.fao.org/3/a-i2698b.pdf accessed August 2019.

31. Public Health England. Composition of foods integrated dataset (CoFID). Available from: https://www.gov.uk/government/publications/composition-of-foods-integrated-dataset-cofid accessed August 2019.

32. Foster E, Hawkins A, Barton KL, et al. Development of food photographs for use with children aged 18 months to 16 years: Comparison against weighed food diaries–The Young Person's Food Atlas (UK). *PloS ONE* 2017;12:e0169084.

33. Foster E, Matthews J, Lloyd J, et al. Children's estimates of food portion size: the development and evaluation of three portion size assessment tools for use with children. *Br J Nutr* 2008;99:175-84.

34. Stevens K. CHU-9D. Measuring health and calculating QALYs for children and adolescents. 2019. Available from: https://licensing.sheffield.ac.uk/i/health-outcomes/CHU-9D.html accessed December 2019.

35. Maguire M, Braun A, Ball S. 'Where you stand depends on where you sit': the social construction of policy enactments in the (English) secondary school. *Discourse: Studies in the Cultural Politics of Education* 2015;36:485-99.

36. Gale NK, Heath G, Cameron E, et al. Using the framework method for the analysis of qualitative data in multi-disciplinary health research. *BMC Med Res Methodol* 2013;13:117.

For peer review only

Figure 1: Logic model and theory of change for the influence of school food standards on children's dietary intake and health outcomes

Supplementary File 1: Recruitment and data collection flow chart

For peer review only - http://bmjopen.bmj.com/site/about/guidelines.xhtml

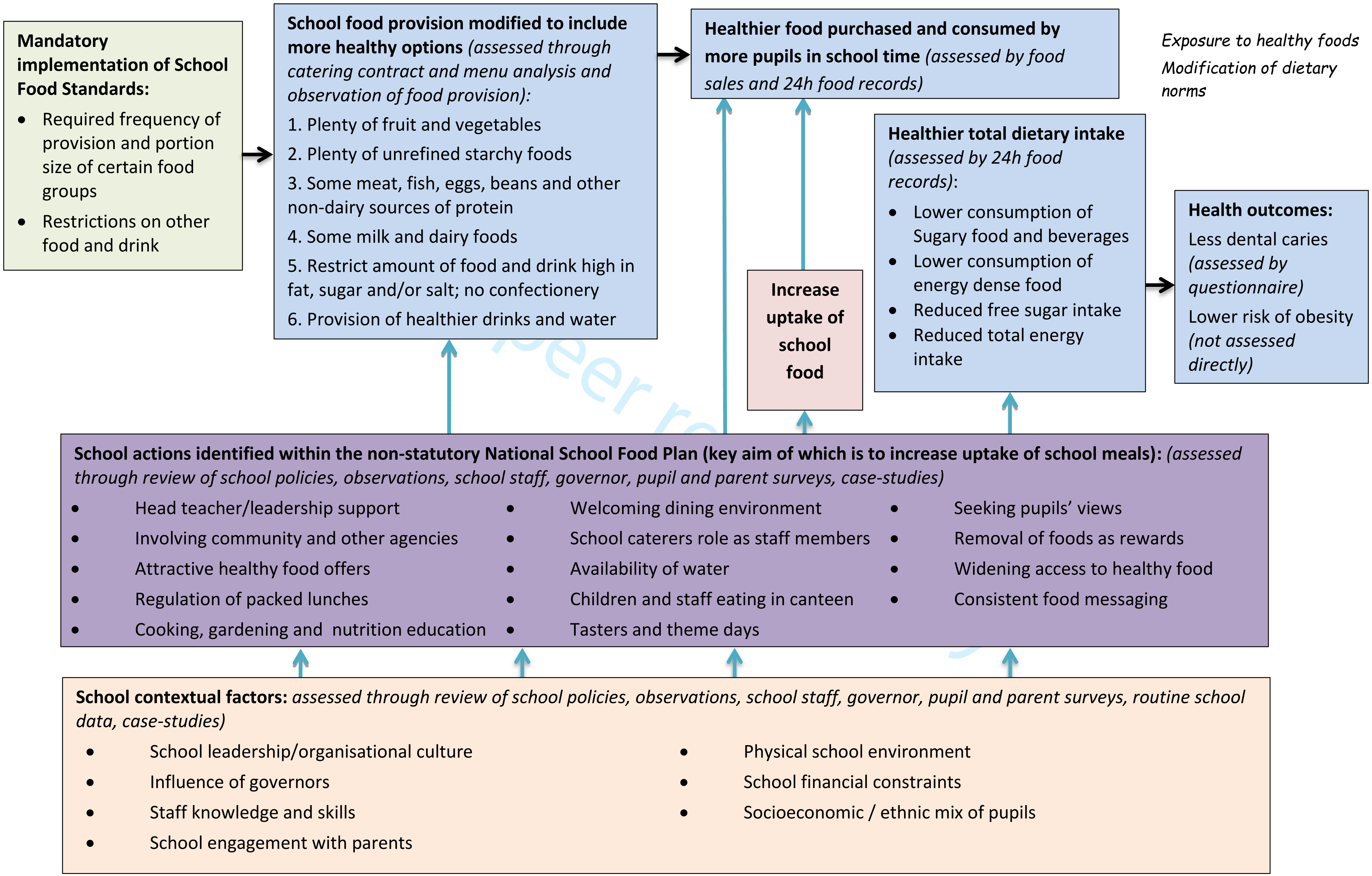
Mandatory implementation of School Food Standards:
Required frequency of provision and portion size of certain food groups
Restrictions on other food and drink
School food provision modified to include more healthy options (assessed through catering contract and menu analysis and observation of food provision):
1. Plenty of fruit and vegetables
2. Plenty of unrefined starchy foods
3. Some meat, fish, eggs, beans and other non-dairy sources of protein
4. Some milk and dairy foods
5. Restrict amount of food and drink high in fat, sugar and/or salt; no confectionery
6. Provision of healthier drinks and water
Healthier food purchased and consumed by more pupils in school time (assessed by food sales and 24h food records)
Exposure to healthy foods
Modification of dietary norms
Healthier total dietary intake (assessed by 24h food records):
Lower consumption of Sugary food and beverages
Lower consumption of energy dense food
Reduced free sugar intake
Reduced total energy intake
Increase uptake of school food
Health outcomes:
Less dental caries (assessed by questionnaire)
Lower risk of obesity (not assessed directly)
School actions identified within the non-statutory National School Food Plan (key aim of which is to increase uptake of school meals): (assessed through review of school policies, observations, school staff, governor, pupil and parent surveys, case-studies)
Head teacher/leadership support
Involving community and other agencies
Attractive healthy food offers
Regulation of packed lunches
Cooking, gardening and nutrition education
Welcoming dining environment
School caterers role as staff members
Availability of water
Children and staff eating in canteen
Tasters and theme days
Seeking pupils' views
Removal of foods as rewards
Widening access to healthy food
Consistent food messaging
School contextual factors: assessed through review of school policies, observations, school staff, governor, pupil and parent surveys, routine school data, case-studies)
School leadership/organisational culture
Influence of governors
Staff knowledge and skills
School engagement with parents
Physical school environment
School financial constraints
Socioeconomic / ethnic mix of pupils

Recruitment and data collection flowchart

RECRUITMENT

SCHOOL

Email: School invitation letters sent to school by email and hard copy

⇩ >7 DAYS

Telephone call: Telephone call to follow up with schools on the email invitation

⇩

Email: Send contract to interested schools prior to initial visit

⇩

Recruitment visit: Attend school to complete recruitment process—sign school contract, identify study liaison, agree a sampling approach for classes with schools, identify difficult times in the school term and agree timeframe, discuss study promotion through social media channels

⇩

PUPIL/STAFF

Parents: Send parent information sheet and opt-out form (hard copy, email or text) to parents of year 7, 9 and 10 pupils

Pupils: Teachers distribute pupil information sheet to year 7, 9 and 10 pupils (hard copy)

⇩ >7 DAYS

CONSENT AND ASSENTTAKING

Opt out: Collect parental opt-out consent forms collected through study liaison staff member

DATA COLLECTION

Staff and Governors: Send staff information sheet, consent form and survey (hard copy and online)

⇩

Request school data: Key information survey, costings survey and other school data and documents e.g. pupil ethnicity data, relevant curriculum documents, school menus

Pupil data collection (session 1) and food services observations visit(s): Attend school— observation of all school food service and dining areas across all eating occasions. Pupil data collection session 1: pupil assent, survey 1 and Intake24 collection 1 with all 3 year groups

Parent survey: Consent form and survey hard copy sent home with pupils at session 1 and online survey link emailed by school

⇩ >7 DAYS

Pupil data collection session 2: Pupil survey 2 and Intake24 collection 2 (staff-led; no researcher present)

Parent survey reminder: School sends reminder via email or text system

Staff survey reminder: Research team sends reminder via email

Vouchers: Pupil vouchers distributed via school following session 2. Parent vouchers sent via post >2 weeks after second reminder.

For peer review only - http://bmjopen.bmj.com/site/about/guidelines.xhtml

SPIRIT 2013 Checklist: Recommended items to address in a clinical trial protocol and related documents*

Section/item	Item No	Description	Addressed on page number
**Administrative information**			
Title	1	Descriptive title identifying the study design, population, interventions, and, if applicable, trial acronym	1
Trial registration	2a	Trial identifier and registry name. If not yet registered, name of intended registry	2 & 3
	2b	All items from the World Health Organization Trial Registration Data Set	1-15
Protocol version	3	Date and version identifier	2
Funding	4	Sources and types of financial, material, and other support	15
Roles and responsibilities	5a	Names, affiliations, and roles of protocol contributors	1 & 15
	5b	Name and contact information for the trial sponsor	14
	5c	Role of study sponsor and funders, if any, in study design; collection, management, analysis, and interpretation of data; writing of the report; and the decision to submit the report for publication, including whether they will have ultimate authority over any of these activities	15
	5d	Composition, roles, and responsibilities of the coordinating centre, steering committee, endpoint adjudication committee, data management team, and other individuals or groups overseeing the trial, if applicable (see Item 21a for data monitoring committee)	14

For peer review only

Introduction

Background and rationale	6a	Description of research question and justification for undertaking the trial, including summary of relevant studies (published and unpublished) examining benefits and harms for each intervention	5-6
	6b	Explanation for choice of comparators	6
Objectives	7	Specific objectives or hypotheses	6
Trial design	8	Description of trial design including type of trial (eg, parallel group, crossover, factorial, single group), allocation ratio, and framework (eg, superiority, equivalence, noninferiority, exploratory)	6

Methods: Participants, interventions, and outcomes

Study setting	9	Description of study settings (eg, community clinic, academic hospital) and list of countries where data will be collected. Reference to where list of study sites can be obtained	6
Eligibility criteria	10	Inclusion and exclusion criteria for participants. If applicable, eligibility criteria for study centres and individuals who will perform the interventions (eg, surgeons, psychotherapists)	7-8
Interventions	11a	Interventions for each group with sufficient detail to allow replication, including how and when they will be administered	N/A
	11b	Criteria for discontinuing or modifying allocated interventions for a given trial participant (eg, drug dose change in response to harms, participant request, or improving/worsening disease)	N/A
	11c	Strategies to improve adherence to intervention protocols, and any procedures for monitoring adherence (eg, drug tablet return, laboratory tests)	10
	11d	Relevant concomitant care and interventions that are permitted or prohibited during the trial	N/A
Outcomes	12	Primary, secondary, and other outcomes, including the specific measurement variable (eg, systolic blood pressure), analysis metric (eg, change from baseline, final value, time to event), method of aggregation (eg, median, proportion), and time point for each outcome. Explanation of the clinical relevance of chosen efficacy and harm outcomes is strongly recommended	10-11

For peer review only - http://bmjopen.bmj.com/site/about/guidelines.xhtml

Participant timeline	13	Time schedule of enrolment, interventions (including any run-ins and washouts), assessments, and visits for participants. A schematic diagram is highly recommended (see Figure)	8, 10, Supplementary file 1
Sample size	14	Estimated number of participants needed to achieve study objectives and how it was determined, including clinical and statistical assumptions supporting any sample size calculations	7
Recruitment	15	Strategies for achieving adequate participant enrolment to reach target sample size	7-8
**Methods: Assignment of interventions (for controlled trials) – N/A**			
Allocation:			
Sequence generation	16a	Method of generating the allocation sequence (eg, computer-generated random numbers), and list of any factors for stratification. To reduce predictability of a random sequence, details of any planned restriction (eg, blocking) should be provided in a separate document that is unavailable to those who enrol participants or assign interventions	N/A
Allocation concealment mechanism	16b	Mechanism of implementing the allocation sequence (eg, central telephone; sequentially numbered, opaque, sealed envelopes), describing any steps to conceal the sequence until interventions are assigned	N/A
Implementation	16c	Who will generate the allocation sequence, who will enrol participants, and who will assign participants to interventions	N/A
Blinding (masking)	17a	Who will be blinded after assignment to interventions (eg, trial participants, care providers, outcome assessors, data analysts), and how	N/A
	17b	If blinded, circumstances under which unblinding is permissible, and procedure for revealing a participant's allocated intervention during the trial	N/A
**Methods: Data collection, management, and analysis**			
Data collection methods	18a	Plans for assessment and collection of outcome, baseline, and other trial data, including any related processes to promote data quality (eg, duplicate measurements, training of assessors) and a description of study instruments (eg, questionnaires, laboratory tests) along with their reliability and validity, if known. Reference to where data collection forms can be found, if not in the protocol	8-11

For peer review only - http://bmjopen.bmj.com/site/about/guidelines.xhtml

	18b	Plans to promote participant retention and complete follow-up, including list of any outcome data to be collected for participants who discontinue or deviate from intervention protocols	10-11
Data management	19	Plans for data entry, coding, security, and storage, including any related processes to promote data quality (eg, double data entry; range checks for data values). Reference to where details of data management procedures can be found, if not in the protocol	11, 13, 14
Statistical methods	20a	Statistical methods for analysing primary and secondary outcomes. Reference to where other details of the statistical analysis plan can be found, if not in the protocol	12
	20b	Methods for any additional analyses (eg, subgroup and adjusted analyses)	12
	20c	Definition of analysis population relating to protocol non-adherence (eg, as randomised analysis), and any statistical methods to handle missing data (eg, multiple imputation)	12
**Methods: Monitoring**			
Data monitoring	21a	Composition of data monitoring committee (DMC); summary of its role and reporting structure; statement of whether it is independent from the sponsor and competing interests; and reference to where further details about its charter can be found, if not in the protocol. Alternatively, an explanation of why a DMC is not needed	N/A
	21b	Description of any interim analyses and stopping guidelines, including who will have access to these interim results and make the final decision to terminate the trial	N/A
Harms	22	Plans for collecting, assessing, reporting, and managing solicited and spontaneously reported adverse events and other unintended effects of trial interventions or trial conduct	N/A
Auditing	23	Frequency and procedures for auditing trial conduct, if any, and whether the process will be independent from investigators and the sponsor	N/A
**Ethics and dissemination**			
Research ethics approval	24	Plans for seeking research ethics committee/institutional review board (REC/IRB) approval	14

For peer review only - http://bmjopen.bmj.com/site/about/guidelines.xhtml

Protocol amendments	25	Plans for communicating important protocol modifications (eg, changes to eligibility criteria, outcomes, analyses) to relevant parties (eg, investigators, REC/IRBs, trial participants, trial registries, journals, regulators)	14
Consent or assent	26a	Who will obtain informed consent or assent from potential trial participants or authorised surrogates, and how (see Item 32)	7-8
	26b	Additional consent provisions for collection and use of participant data and biological specimens in ancillary studies, if applicable	N/A
Confidentiality	27	How personal information about potential and enrolled participants will be collected, shared, and maintained in order to protect confidentiality before, during, and after the trial	14
Declaration of interests	28	Financial and other competing interests for principal investigators for the overall trial and each study site	15-16
Access to data	29	Statement of who will have access to the final trial dataset, and disclosure of contractual agreements that limit such access for investigators	N/A
Ancillary and post-trial care	30	Provisions, if any, for ancillary and post-trial care, and for compensation to those who suffer harm from trial participation	N/A
Dissemination policy	31a	Plans for investigators and sponsor to communicate trial results to participants, healthcare professionals, the public, and other relevant groups (eg, via publication, reporting in results databases, or other data sharing arrangements), including any publication restrictions	14
	31b	Authorship eligibility guidelines and any intended use of professional writers	N/A
	31c	Plans, if any, for granting public access to the full protocol, participant-level dataset, and statistical code	14
**Appendices**			
Informed consent materials	32	Model consent form and other related documentation given to participants and authorised surrogates	
Biological specimens	33	Plans for collection, laboratory evaluation, and storage of biological specimens for genetic or molecular analysis in the current trial and for future use in ancillary studies, if applicable	N/A

For peer review only - http://bmjopen.bmj.com/site/about/guidelines.xhtml

*It is strongly recommended that this checklist be read in conjunction with the SPIRIT 2013 Explanation & Elaboration for important clarification on the items. Amendments to the protocol should be tracked and dated. The SPIRIT checklist is copyrighted by the SPIRIT Group under the Creative Commons "Attribution-NonCommercial-NoDerivs 3.0 Unported" license.

For peer review only

For peer review only - http://bmjopen.bmj.com/site/about/guidelines.xhtml
