# Supplementary material for: The Food provision, cUlture and Environment in secondary schooLs (FUEL) study: protocol of a mixed methods evaluation of national School Food Standards implementation in secondary schools and their impact on pupils’ dietary intake and dental health
Source: BMJ Open. 2020 Oct 16;10(10):e042931. doi: 10.1136/bmjopen-2020-042931 (PMC7569925; doi:10.1136/bmjopen-2020-042931)
Supplement: Reviewer comments [file bmjopen-2020-042931.reviewer_comments.pdf]

PEER REVIEW HISTORY

BMJ Open publishes all reviews undertaken for accepted manuscripts. Reviewers are asked to complete a checklist review form (**http://bmjopen.bmj.com/site/about/resources/checklist.pdf**) and are provided with free text boxes to elaborate on their assessment. These free text comments are reproduced below.

ARTICLE DETAILS

**TITLE (PROVISIONAL)**	The Food provision, cUlture and Environment in secondary schooLs (FUEL) study: protocol of a mixed methods evaluation of national School Food Standards implementation in secondary schools and their impact on pupils' dietary intake and dental health
**AUTHORS**	Murphy, Marie; Pallan, Miranda; Lancashire, Emma; Duff, Rhona; Adamson, Ashley; Bartington, Suzanne; Frew, Emma; Griffin, Tania; Hurley, Kiya; Parry, Jayne; Passmore, S; Ravaghi, Vahid; Sitch, Alice; Spence, Suzanne; Rowland, Maisie; Wheeldon, Scott; Adab, Peymane

This article was not externally reviewed at BMJ Open. Protocols that have been independently assessed prior to submission to BMJ Open are usually fast-tracked to publication on the grounds that further substantial changes will not be possible. This independent assessment will usually be external, independent review for both a substantial grant award from a non-commercial or government funder and ethics approval.
